# Zone equalisation normalisation for improved alignment of epigenetic signal

**DOI:** 10.1093/bioinformatics/btag622

**Published:** 2026-08-20

**Authors:** Tom Wilson, Thomas A Milne, Simone G Riva, Jim R Hughes

**Affiliations:** MRC Molecular Haematology Unit, MRC Weatherall Institute of Molecular Medicine, University of Oxford, Oxford, United Kingdom; MRC WIMM Centre for Computational Biology, MRC Weatherall Institute of Molecular Medicine, University of Oxford, Oxford, United Kingdom; MRC Molecular Haematology Unit, MRC Weatherall Institute of Molecular Medicine, University of Oxford, Oxford, United Kingdom; MRC WIMM Centre for Computational Biology, MRC Weatherall Institute of Molecular Medicine, University of Oxford, Oxford, United Kingdom; MRC Molecular Haematology Unit, MRC Weatherall Institute of Molecular Medicine, University of Oxford, Oxford, United Kingdom; MRC WIMM Centre for Computational Biology, MRC Weatherall Institute of Molecular Medicine, University of Oxford, Oxford, United Kingdom; MRC Molecular Haematology Unit, MRC Weatherall Institute of Molecular Medicine, University of Oxford, Oxford, United Kingdom; MRC WIMM Centre for Computational Biology, MRC Weatherall Institute of Molecular Medicine, University of Oxford, Oxford, United Kingdom

## Abstract

**Motivation:**

High-throughput genomic technologies have transformed our understanding of biological systems, yet direct comparison and visualisation of these complex datasets remains challenging. Existing normalisation methods often fail to align genomic signal across samples due to sensitivity to sequencing depth differences and localised high-signal artefacts, leading to inconsistent replicate behaviour and increased downstream variability.

**Results:**

We introduce Zone Equalisation Normalisation (ZEN), a novel approach designed to improve cross-sample signal alignment of genomic data. ZEN rescales genomic signal based on variance estimated within biologically enriched regions, reducing the influence of extreme outliers while preserving underlying biological structure. Using a diverse collection of data and our new genome-wide benchmarking approach, we reveal that ZEN improves biological and technical replicate alignment across the majority of tested conditions and experimental platforms. We further show that this improved signal comparability is associated with fewer differential accessibility calls between technical replicates and a more conservative set of biological differences. Together, these results demonstrate that ZEN provides a complementary framework to improve the accuracy and reliability of genomic data analysis and that normalisation choice can affect downstream analyses and biological interpretation.

**Availability and implementation:**

ZEN is available as an open-source Python package via conda and PyPI. Source code, documentation, tutorials, and code to reproduce the analyses are available at https://github.com/Genome-Function-Initiative-Oxford/Zone-Equalisation-Normalisation and Zenodo (https://doi.org/10.5281/zenodo.21067751).

## 1 Introduction

High-throughput sequencing-based assays have revolutionised our understanding of gene regulation by enabling comprehensive interrogation of molecular mechanisms. Assay for transposase-accessible chromatin using sequencing (ATAC-seq) provides insights into chromatin accessibility, which acts as a proxy to identify active enhancers and promoters (Buenrostro *et al*. 2015); chromatin immunoprecipitation followed by sequencing (ChIP-seq) reveals protein-DNA interactions like transcription factors (TFs) and histone modifications (Barski *et al*. 2007, Johnson *et al*. 2007); and transient transcriptome sequencing (TT-seq) captures nascent transcription at unprecedented resolution (Park 2009, Buenrostro *et al*. 2013, Schwalb *et al*. 2016). Raw data generated by these technologies contain both biological and technical variation. Yet the latter can significantly compromise accuracy and reliability of scientific interpretation (Bullard *et al*. 2010, Dillies *et al*. 2013).

Normalisation is a critical preprocessing step in genomic data analysis to scale signal tracks for direct, meaningful comparison across samples. The goal is to address systematic biases and technical artefacts that distort cross-sample signal comparability and obscure genuine biological signals (Robinson *et al*. 2010, Hafemeister and Satija 2019). Technical variation can arise from sequencing depth differences, batch effects, sample preparation methodologies, instrument-specific technical noise, genomic content variations and read mapping issues (Leek *et al*. 2010, McCarthy *et al*. 2012). Biological variation reflects genuine biological changes across cell types, tissues, developmental stages and environmental conditions.

Many normalisation methods have been developed to address challenges in genomic data visualisation and cross-sample comparisons, namely those across multiple biological or technical samples. For visualisation, global normalisation techniques are most commonly used, such as reads per kilobase per million mapped reads (RPKM), counts per million (CPM), bins per million mapped reads (BPM) and reads per genome coverage (RPGC), which adjust sequencing depth, total mapped reads, raw counts, and genome-wide coverage, respectively (Mortazavi *et al*. 2008, Wagner *et al*. 2012). However, these methods often fail to adequately align samples across experiments as their reliance on library size does not account for compositional biases and is highly sensitive to factors such as elevated mitochondrial read fractions (Zhao *et al*. 2020).

For ChIP-seq, matched input controls that measure background to address sequencing bias can also be used to scale signal across samples (Liang and Keleş 2012). However, they are often not generated per replicate limiting their use. Similarly, signal can be rescaled using spike-in exogenous chromatin (ATAC-seq and ChIP-seq) or exogenous RNA (TT-seq) as a stable reference, often by reference-adjusted reads per million (RRPM) (Orlando *et al*. 2014, Schwalb *et al*. 2016, Patel *et al*. 2024). Although this is considered a standard for TT-seq, it requires additional experimental steps that are often not comparable across experiments.

Within DiffBind, a popular tool designed for ChIP-seq differential binding (DB) and ATAC-seq differential accessibility (DA) analysis, the default normalisation method is full library size (LIB) in which scaling factors are derived from the total number of aligned reads per replicate (Ross-Innes *et al*. 2012). DiffBind also supports edgeR’s trimmed mean of M-values (TMM) and DESeq2’s relative log expression (RLE) normalisation (Robinson *et al*. 2010, Love *et al*. 2014). These more sophisticated methods account for compositional differences and systematic biases across samples, but require batch processing of samples, limiting their flexibility. The applicability of these between-sample methods for bigWig scaling is unclear as these were designed to scale discrete RNA-seq count matrices rather than continuous signal profiles. As each normalisation strategy offers unique advantages and limitations, careful selection is required based on experimental design, data characteristics, and specific research objectives.

To address these limitations, we introduce a new normalisation method, Zone Equalisation Normalisation (ZEN), that equalises variance within biologically enriched regions while remaining robust to extreme technical artefacts, to improve cross-sample signal alignment. This builds upon the normalisation method introduced by Riva *et al.* for training an ML model, REnformer, to predict chromatin accessibility (Riva *et al*. 2025). We demonstrate using ATAC-seq, ChIP-seq and TT-seq how ZEN directly operates on bigWig genomic signal tracks and is therefore applicable to multiple assays. Furthermore, we present a new genome-wide approach to benchmark normalisation methods, show how ZEN can be incorporated into DA analysis to reduce false positives and outline a novel approach to recreate pre-normalised bigWigs.

## 2 Materials and methods

### 2.1 ZEN workflow

**Figure 1:**
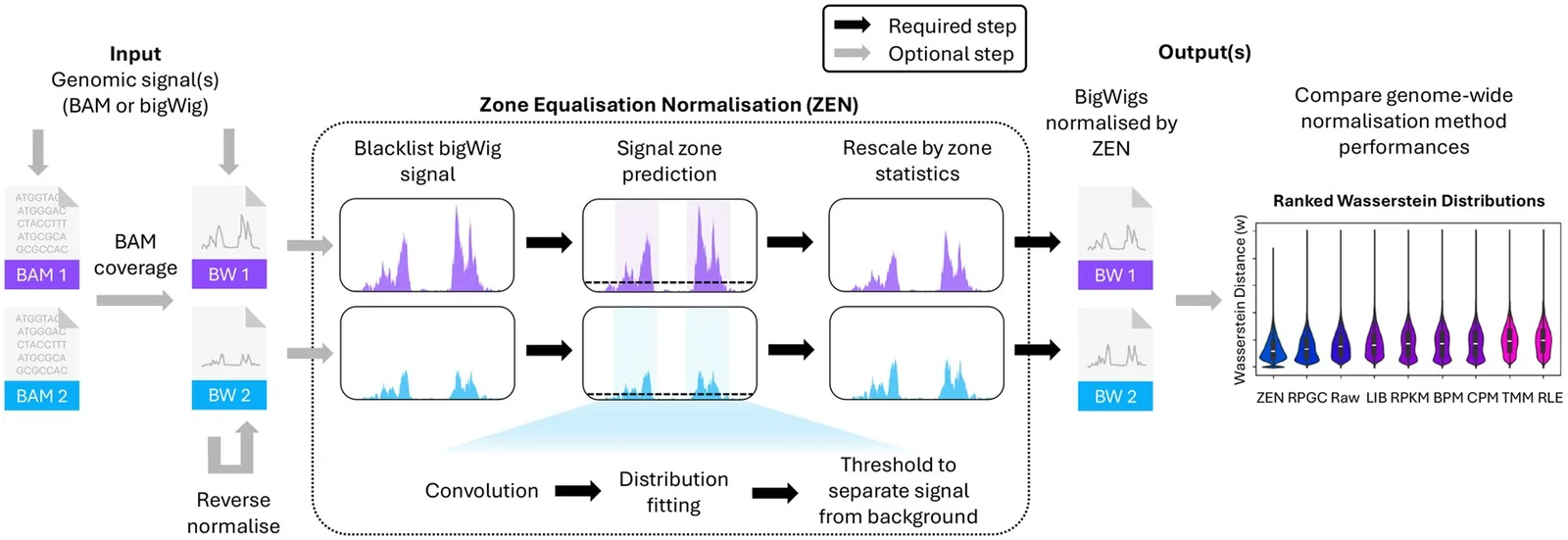
Graphical overview of ZEN. Genomic signal per sample is inputted from either BAM or bigWig. If BAMs are given, these are converted to bigWig, while if bigWigs are directly given, these can optionally be reverse normalised. BigWig signal is then blacklisted, and signal zones predicted, which involves signal smoothing via convolution, distribution fitting and chromosome-specific thresholding to separate signal of interest from background noise. BigWigs normalised with ZEN are outputted, and alignment across samples may optionally be compared against other normalisation methods through Wasserstein distance plots.

Figure 1 illustrates the complete ZEN workflow, which processes genomic signals through several key stages including: blacklist filtering, signal convolution, distribution fitting, signal zone prediction, quality filtering, and signal rescaling to produce normalised bigWigs.

#### 2.1.1 Blacklisting

Blacklisting is a recommended quality control step to remove known regions of problematic signal, such as repetitive regions that aligners cannot reliably map to. Considering a set of chromosomes C, the unnormalised signal is defined as $y_{c}=y_{c_{1}},\ldots y_{c_{n}}$, where c∈C and *n* is the size of chromosome *c*. To begin, blacklisted coordinates are excluded from the original signal $y_{c}$, which is read from the input BAM (mapped with deepTools bamCoverage (Ramírez *et al*. 2014)) or bigWig.

#### 2.1.2 Signal convolution

As assays such as ATAC-seq are prone to signal fluctuations, such as spikes due to random technical noise, or disruptions in alignment, like indels, a smoothed version of the signal is created to improve consistency in signal detection across genomic regions. This is only used to estimate normalisation parameters, while ZEN normalised bigWigs are made from scaling the original signal.

Before convolution, regions with 500 base pairs (bp) or more of consecutive zeros are masked for efficiency, creating an intermediate signal ${\bar{y}}_{c}={\bar{y}}_{c_{1}},\ldots ,{\bar{y}}_{c_{n}}$. A triangular kernel ${\hat{\kappa }}_{n}$ is created of size n=301 bp (Eq. 1), then normalised to obtain $\kappa_{n}$ (Eq. 2).


(1) $${\hat{\kappa }}_{n_{j}}=\{\begin{array}{cc} j+1, & 0\leq j<\left\lceil \frac{n}{2}\right\rceil \\ n-j, & \left\lfloor \frac{n}{2}\right\rfloor \leq j<n \end{array}$$

(2) $$\kappa_{n_{j}}=\frac{{\hat{\kappa }}_{n_{j}}}{\sum_{j}{\hat{\kappa }}_{n_{j}}}$$

Convolution is performed on ${\bar{y}}_{c}$ using $\kappa_{n}$ (Eq. 2.1.2), creating smoothed signal ${\tilde{y}}_{c}={\tilde{y}}_{c_{1}},\ldots ,{\tilde{y}}_{c_{n}}$. For regions shorter than *n*, the kernel is symmetrically trimmed to fit the region. Although both the shape and size *n* of the kernel are customisable (Supplementary Methods B.3, available as supplementary data at *Bioinformatics* online), $\kappa_{301}$ was chosen as the default to fit the typical size range of regulatory element (RE) peaks.


(3) $${\tilde{y}}_{c_{i}}=({\bar{y}}_{c}*\kappa )(i)=\sum_{j=-\left\lceil \frac{n}{2}\right\rceil }^{\left\lfloor \frac{n}{2}\right\rfloor }{\bar{y}}_{c_{i-j}}\cdot \kappa_{n_{j}}$$

#### 2.1.3 Distribution fitting

Before zone prediction, a parametric distribution is fitted per chromosome to approximate the empirical distribution of the smoothed signal (after transformation explained below). The aim is to derive a sample-specific quantile-based threshold from this fitted distribution for the initial separation of signal regions from background noise.

Zeros are removed from ${\tilde{y}}_{c}$, and its absolute values are log-transformed to improve symmetry and model it as a continuous statistical distribution (Supplementary Methods B.1, available as supplementary data at *Bioinformatics* online). This creates the transformed smooth signal, $y_{c}^{\prime }=y_{c_{1}}^{\prime },\ldots ,y_{c_{n-z}}^{\prime }$, where *z* is the number of zeros for chromosome *c* (Eq. 3).


(4) $$y_{c}^{\prime }=[log_{2}(|{\tilde{y}}_{c_{i}}|),\text{if}\;y_{c_{i}}>0,i\in [1,\ldots ,n]]$$

To reduce run time, memory and over-fitting, $y_{c}^{\prime }$ is down-sampled to δ positions (Eq. 4), forming ${\tilde{y}}_{c}^{\prime }={\tilde{y}}_{c_{1}}^{\prime },\ldots ,{\tilde{y}}_{c_{\delta }}^{\prime }$, using a seed for reproducibility across runs. By default, δ=300 was chosen to provide a stable approximation of the data without requiring tuning while maintaining sensitivity in distribution fitting. The effect of changing δ is explored in Supplementary Methods B.3, available as supplementary data at *Bioinformatics* online. Though down-sampling can be disabled to make ZEN fully deterministic.


(5) $${\tilde{y}}_{c}^{\prime }\subset y_{c}^{\prime },|{\tilde{y}}_{c}^{\prime }|=\delta ,\;\delta <n$$

Laplace is the default distribution as it provides a robust approximation of the empirical signal distribution of $y_{c}^{\prime }$ for different data types (Supplementary Methods B.1, available as supplementary data at *Bioinformatics* online). By default, scale $V_{c}$ (Eq. 5) is based on the median absolute deviation (MAD) from the median: $\text{MAD}(x)=\text{median}(|x_{i}-\text{median}(x)|)$, or mean average deviation (MeanAD) if MAD is zero: $\text{MeanAD}(x)=\text{mean}|x_{i}-\text{mean}(x)|$. For d=Laplace, constants are set as $\alpha_{d}\approx 1.4427$ and $\beta_{d}=1$ (see Supplementary Methods Table S1, available as supplementary data at *Bioinformatics* online for distribution-specific constants).


(6) $$V_{c}=\{\begin{array}{cc} \alpha_{d}\cdot \text{MAD}({\tilde{y}}_{c}^{\prime }) & \text{if MAD }({\tilde{y}}_{c}^{\prime })\neq 0\\ \beta_{d}\cdot \text{MeanAD}({\tilde{y}}_{c}^{\prime }), & \text{otherwise} \end{array}$$

#### 2.1.4 Signal zone prediction and quality filtering

A key step in ZEN is to identify biologically enriched signal regions, termed zones, from which scaling factors based on signal variance are derived. Zone prediction is a multi-step, adaptive and data-driven procedure consisting of thresholding, quality filtering and padding. It does not aim to optimise boundaries at base-pair resolution or replace dedicated peak-callers, but to identify regions for robust variance estimation.

Initial zone boundaries are estimated using the zone probability parameter, p∈(0,1). This is the selected cumulative probability level (quantile) of the fitted distribution at which to separate signal from background noise. For example, p=0.995 (the default) sets a cut-off between the lower 99.5% and upper 0.5% of the modelled signal. Using the fitted distribution’s percentage point function (PPF) (Johnson *et al*. 1995), *p* is translated to a zone threshold $\lambda_{c}$ (Eq. 6) to set a cut-off based on the data. See Supplementary Methods B.3, available as supplementary data at *Bioinformatics* online for the effect of adjusting *p*.


(7) $$\lambda_{c}=\{\begin{array}{cc} e^{\text{PPF}(p)}, & \text{if}\;y_{c}\;\text{is}\;\text{positive}\\ -e^{\text{PPF}(p)}, & \text{if}\;y_{c}\;\text{is}\;\text{negative} \end{array}$$

The smoothed signal, ${\tilde{y}}_{c}$, is thresholded using $\lambda_{c}$ and positions exceeding it consecutively for a minimum region size *r* bp or more (or below for negative signal) are set as unfiltered zones (Eq. 7). The default, r=35, was chosen to balance finding sufficient signal with capturing short motifs (Supplementary Methods B.2 and B.3, available as supplementary data at *Bioinformatics* online).


(8) $$\begin{array}{c} t_{c}=\{|{\tilde{y}}_{c_{i}}|>|\lambda_{c}|,\;i\in [1,\ldots ,n]\},\;s.t.\;t_{c}\subseteq [1,\ldots ,n]\\ {z^{\prime }}_{c}=\left\{(\varsigma ,\varepsilon )|\begin{array}{c} \forall x\in t_{c},\\ \varsigma \leq x\leq \varepsilon \;\wedge \\ ∄\;(x\in t_{c}|x=\varepsilon +1)\;\wedge \\ ∄\;(x\in t_{c}|x=\varsigma -1)\;\wedge \\ |\varepsilon -\varsigma +1|\geq r \end{array}\right\} \end{array}$$

Before filtering zones to remove low quality signal, the estimated fragment size $\hat{f_{c}}$ (Eq. 8), mean of non-zero signal per chromosome $\mu_{c}$ and the global non-zero mean across the genome $\mu_{g}$ (Eq. 9) are calculated. $\hat{f_{c}}$ is a proxy for the value generated by a single sequenced read per chromosome.


(9) $${\hat{f}}_{c}=\{\begin{array}{cc} \min(\{y_{c_{i}}|y_{c_{i}}\neq 0, & i\in [1,\ldots ,n]\})\;\text{if}y_{c}\text{ispositive}\\ \max(\{y_{c_{i}}|y_{c_{i}}\neq 0, & i\in [1,\ldots ,n]\})\;\text{if}y_{c}\text{isnegative} \end{array}$$

(10) $$\mu_{c}=\text{mean}(\{y_{c_{i}}|y_{c_{i}}\neq 0\});\;\mu_{g}=\text{mean}\left(\left[\underset{c\in C}{\cup }y_{c_{i}}|y_{c_{i}}\neq 0\right]\right)$$

The quality filter threshold, $\tau_{c}$, is defined as the maximum (for positive signal) or minimum (for negative signal) of the scaled estimated fragment size and the non-zero means (Eq. 10).


(11) $$\tau_{c}=\{\begin{array}{cc} \text{max}(10\hat{f},1.5\mu_{c},1.5\mu_{g}), & \text{if}\;y_{c}\;\text{positive}\;\text{positive}\\ \text{min}(10\hat{f},1.5\mu_{c},1.5\mu_{g}), & \text{if}\;y_{c}\;\text{is}\;\text{negative} \end{array}$$

To refine the zones, coordinates in $z_{c}^{\prime }$ are filtered using the absolute values of the original signal, $\left|y_{c}\right|$, to retain regions where $\left|\tau_{c}\right|$ is exceeded for a minimum *m* bp consecutive differences. This forms unpadded zones (Eq. 11), for which low quality signal and very sparsely mapped chromosomes are excluded. *L* is a subset of indices with different consecutive values between ς and ε. By default, m=5 was found to be a good balance between removing noise and isolated high value artefacts with retaining high quality signal (further explanation in Supplementary Methods B.2 and B.3, available as supplementary data at *Bioinformatics* online).


(12) $$z_{c}^{\prime \prime }=\left\{(\varsigma ,\varepsilon )\;|\begin{array}{cc} & \exists \;L\subseteq z_{c}^{\prime }\;\wedge \\ & |L|\geq m\;\wedge \\ & \forall x\in L,|y_{c_{x}}|>|\tau_{c}| \end{array}\;\right\},$$

Padded zones $z_{c}^{\prime \prime \prime }$ are created from unpadded zones $z_{c}^{\prime \prime }$ by a function $f(z_{c}^{\prime \prime },d,b)$ to merge zones within d=5 bp, add b=1000 to pad the start and end, rounds outwards to the nearest padding bin edge of size *b*, and merge coordinates within *d* bp (Eq. 12). The effect of different values for *d* and *b* are explored in Supplementary Methods B.3, available as supplementary data at *Bioinformatics* online. For a visualisation of zone prediction, see Supplementary Methods Figure S5, available as supplementary data at *Bioinformatics* online.


(13) $$z_{c}^{\prime \prime \prime }=\{(\varsigma ,\varepsilon )|(\varsigma ,\varepsilon )\in f(z_{c}^{\prime \prime },d,b)\}$$

#### 2.1.5 Rescaling signal by zone statistics

ZEN is a form of variance normalisation inspired by RNA-seq z-score normalisation (Zhao *et al*. 2021). It adjusts the signal distribution per sample to achieve a consistent spread across sample-specific zones, thereby avoiding the need for batch processing. Scaling factors can either be derived from padded zones (default) to account for both strongly enriched signal and a small amount of surrounding signal, or unpadded zones to use only strongly enriched signal (Eq. 13 and Supplementary Methods B.3, available as supplementary data at *Bioinformatics* online).


(14) $$\zeta_{c}=\{\begin{array}{c} z_{c}^{\prime \prime \prime }\;\text{if}\;\text{use}\;\text{padded}\;\text{zones}\\ z_{c}^{\prime \prime }\;\text{if}\;\text{use}\;\text{unpadded}\;\text{zones} \end{array}$$

For each c∈C, the signal within the coordinates of each zone $\zeta_{c}$ is extracted as $y_{c}^{z}$ (Eq. 14).


(15) $$y_{c}^{z}=\left[y_{c_{i}}\;|\;i\in [\varsigma ,\ldots ,\varepsilon ],\;(\varsigma ,\varepsilon )\in \zeta_{c}\right]$$

After removing blacklisted regions from the original signal *y*, extreme outliers may remain that can arise from technical artefacts, such as mappability issues, or biological activity such as highly transcribed regions. To exclude these, zeros are removed and the $10^{th}$ lower ($P_{10}$) and upper ($P_{90}$) percentiles are trimmed from $y_{c}^{z}$ creating trimmed signal $y_{c}^{p}$ (Eq. 15).


(16) $$y_{c}^{p}=\left[x_{i}\;|\;x_{i}\in y_{c}^{z},\;x_{i}\neq 0,\;P_{10}<x_{i}<P_{90}\right]$$

To reduce bias from extreme regions, the standard deviation $\sigma_{c}^{p}$ is calculated for each *c* from $y_{c}^{p}$. Then, the median $\sigma_{c}^{p}$ across all c∈C are calculated as $\sigma^{p}$ (Eq. 16).


(17) $$\sigma^{p}=\text{median}\left(\left[\sigma_{c}^{p}\;|\;c\in C\right]\right)$$

ZEN normalised signal is created by dividing the original signal per chromosome ($y_{c}$) by $\sigma^{p}$. This sets variance as approximately 1 within non-zero signal over zones (Eq. 17). Normalised signal per sample is saved to bigWig.


(18) $$\text{ZEN}=\left[\frac{y_{c}}{\sigma^{p}}\;|\;\forall c\in C\right]$$

### 2.2 Wasserstein distance distributions

To quantitatively evaluate genomic signal alignment genome-wide across samples and replicates, we use Wasserstein distance to summarise and rank pairwise signal similarity under different normalisation methods. Wasserstein distance is well suited to genomic signals because it compares the overall shape and spatial distribution of signal while remaining robust to differences in signal magnitude, sequencing depth, and technical noise. Signal regions were first selected and merged pairwise across samples. For punctate assays (i.e. ATAC-seq and ChIP-seq) we used LanceOtron peak calls, whereas for non-punctate assays (i.e. TT-seq) we used zones $z_{c}^{\prime \prime \prime }$, ∀c∈C. Alternatively, other peak callers or custom coordinates can be used.

To account for scale differences between normalisation methods, min-max scaling was performed between pairwise sample signals, producing two normalised, non-negative signal profiles, $r_{1}$ and $r_{2}$, that represent discrete one-dimensional probability distributions. Wasserstein distance *w* (Eq. 18) was then computed, where $\Gamma (r_{1},r_{2})$ denotes the set of all joint probability distributions on R × R with $r_{1}$ and $r_{2}$ as marginals (Villani 2008). Here, $r_{1}(x)$ and $r_{2}(x)$ specify the probability densities at genomic position *x* for each sample. Overall lower *w* indicates stronger alignment.


(19) $$w(r_{1},r_{2})=\underset{\pi \in \Gamma (r_{1},r_{2})}{\text{inf}}\int_{R\times R}|x-y|d\pi (x,y)$$

### 2.3 Code availability

All code developed for this study is open source and accessible to the research community. ZEN is packaged as a Python library and distributed via conda and PyPI. Source code, documentation, tutorials and code to reproduce the analyses of this paper are hosted on GitHub (https://github.com/Genome-Function-Initiative-Oxford/Zone-Equalisation-Normalisation) and Zenodo (https://doi.org/10.5281/zenodo.21067751).

### 2.4 Differential accessibility analysis

To perform DA analysis, DiffBind was run using the default parameters (DESeq2) and LanceOtron peak calls on autosomal and sex chromosomes after blacklisting by ENCODE hg38 v2 (Amemiya *et al*. 2019). Results with Benjamini-Hochberg (BH) adjusted *P*-values less than 0.05 were considered significant.

### 2.5 Data availability

We collected a diverse set of genomic datasets to evaluate different normalisation methods’ performances across sequencing technologies, experimental contexts and cell types. For chromatin accessibility and cohesin binding, ATAC-seq and ChIP-seq data were obtained from day 13 erythroid differentiation experiments. This includes bulk ATAC-seq from two donors (donors 2 and 3), each with seven replicates, deposited in the Gene Expression Omnibus (GEO) under accession code GSE311157, and RAD21 ChIP-seq from three donors (donors 1, 2, and 30), each with three replicates (GSE244929) (Georgiades *et al*. 2025). RAD21 donor 30 rep 2 was excluded due to low sequencing depth. Untreated HeLa cell TT-seq with three replicates (GSE284682) (Kim *et al*. 2025) and HEK293T embryonic kidney cell line with four replicates (GSE218127) (Mimoso and Adelman 2023) were also downloaded.

Data was processed by the CATCH-UP pipeline (Riva *et al*. 2023) with default parameters and reads mapped to human genome hg38 unless otherwise stated. For TT-seq, no read extension was used, and reads were mapped to a SeqNado (https://pypi.org/project/seqnado/) combined hg38 and spike-in RNA genome (sacCer3 for HeLa and dm6 for HEK293T). BigWigs per sample were created without normalisation (raw), and with RPKM, CPM, BPM, and RPGC set at the bamCoverage stage. ZEN normalised bigWigs were generated from raw bigWigs with default parameters unless otherwise stated. The blacklist was set as ENCODE hg38 v2 (Amemiya *et al*. 2019). LIB, RLE and TMM normalised bigWigs were made by scaling raw bigWigs by DiffBind scaling factors, while RRPM normalised bigWigs were created by scaling raw bigWigs by spike-in reads per million. For experiments with narrow peaks (ATAC-seq and RAD21 ChIP-seq), peaks were called with LanceOtron, keeping those with overall peak score >0.5 (Hentges *et al*. 2022).

## 3 Results

### 3.1 ZEN improves sample alignment over functionally important loci

**Figure 2:**
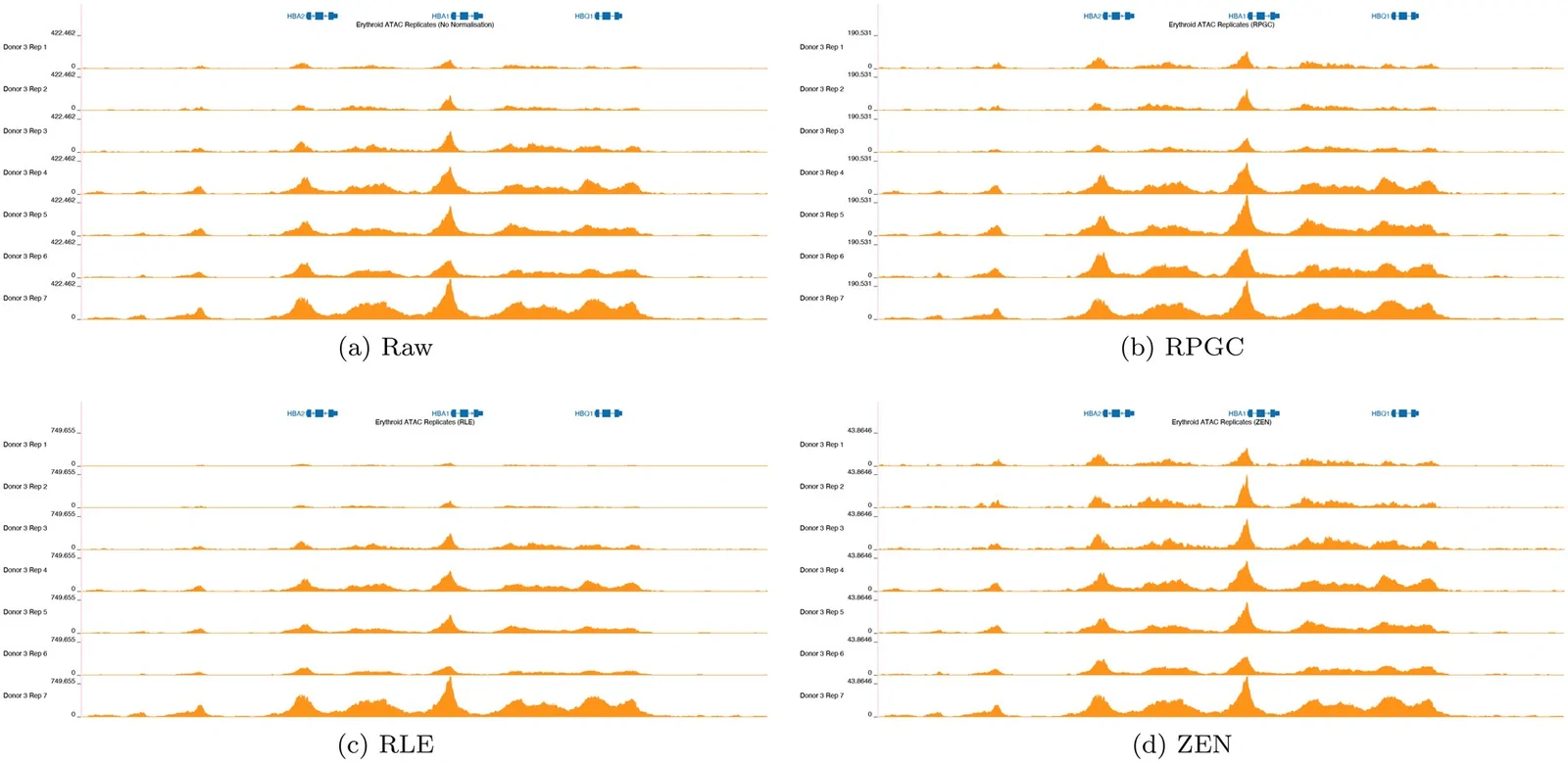
Genome Browser view of erythroid donor 3 ATAC-seq coverage over chromosome 16 (chr16:167 000–185 000) across the alpha-globin *locus* and surrounding genes (*HBM*, *HBA1*, *HBA2*, and *HBQ1*). Tracks show technical reps 1 to 7 without normalisation (a), with RPGC (b), RLE (c) and ZEN (d). For RPKM, CPM, BPM, LIB and TMM, see Figure S8, available as supplementary data at *Bioinformatics* online.

To assess the ability of different normalisation methods to reduce technical variability for ATAC-seq and ChIP-seq, we benchmarked ZEN against raw (unnormalised) signal, RPKM, CPM, BPM, RPGC, LIB, RLE and TMM for the erythroid ATAC-seq (Figure 2 and Supplementary Results Figure S8, available as supplementary data at *Bioinformatics* online) and erythroid RAD21 ChIP-seq (Supplementary Results Figure S9, available as supplementary data at *Bioinformatics* online). At the alpha-globin locus, a well-characterised regulatory locus that produces haemoglobin in healthy erythroid cells (Weiss and Liebhaber 1994), effective normalisation should align enriched signal over biological and technical replicates. Failure to achieve this would introduce artificial variability that can obscure true regulatory signal and compromise downstream analyses. However, only ZEN and RPGC improved sequencing depth differences between replicates for the ATAC-seq, with ZEN being most consistent. In contrast, global library size based methods (RPKM, CPM, BPM) increased misalignment, likely reflecting their sensitivity to technical artefacts that escape blacklisting, such as high-signal regions near centromeres that substantially vary across technical replicates (Supplementary Results Figure S10, available as supplementary data at *Bioinformatics* online). For the RAD21 ChIP-seq, both ZEN and global methods improved locus alignment across replicates. In both assays, between-sample methods (LIB, RLE and TMM) detrimentally increased cross-replicate variability.

**Figure 3:**
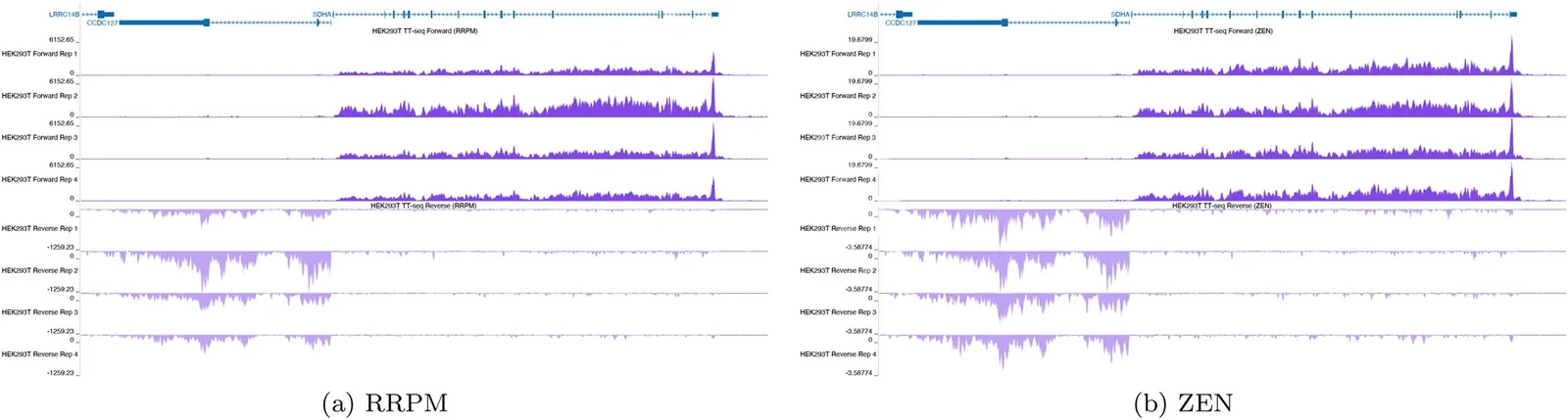
Genome Browser view of HEK293T TT-seq over chromosome 5 (chr5:193 000–262 000). Forward strand transcription is over *CCDC127* and reverse strand transcription over the housekeeping gene *SDHA*. Tracks show technical reps 1 to 4 with RRPM spike-in normalisation (a) and ZEN (b).

To evaluate the HeLa and HEK293T TT-seq, ZEN was benchmarked against raw signal, RPKM, CPM, BPM, RPGC and RRPM. Inspecting the *SDHA* locus, a housekeeping gene with highly stable RNA expression in HeLa cells (Krainova *et al*. 2013), all normalisation methods improved HeLa technical replicate alignment (Supplementary Results Figure S11, available as supplementary data at *Bioinformatics* online), particularly over exonic regions. For HEK293T, it was less clear which method most strongly aligned technical replicate signals. However, RRPM noticeably increased cross-replicate variability (Fig. 3), generating apparent differences where no true biological variation is expected. However, manually assessing TT-seq alignment is challenging due to the broad and variable domains created by transcriptional signal.

### 3.2 ZEN improves genome-wide sample alignment relative to conventional methods

**Figure 4:**
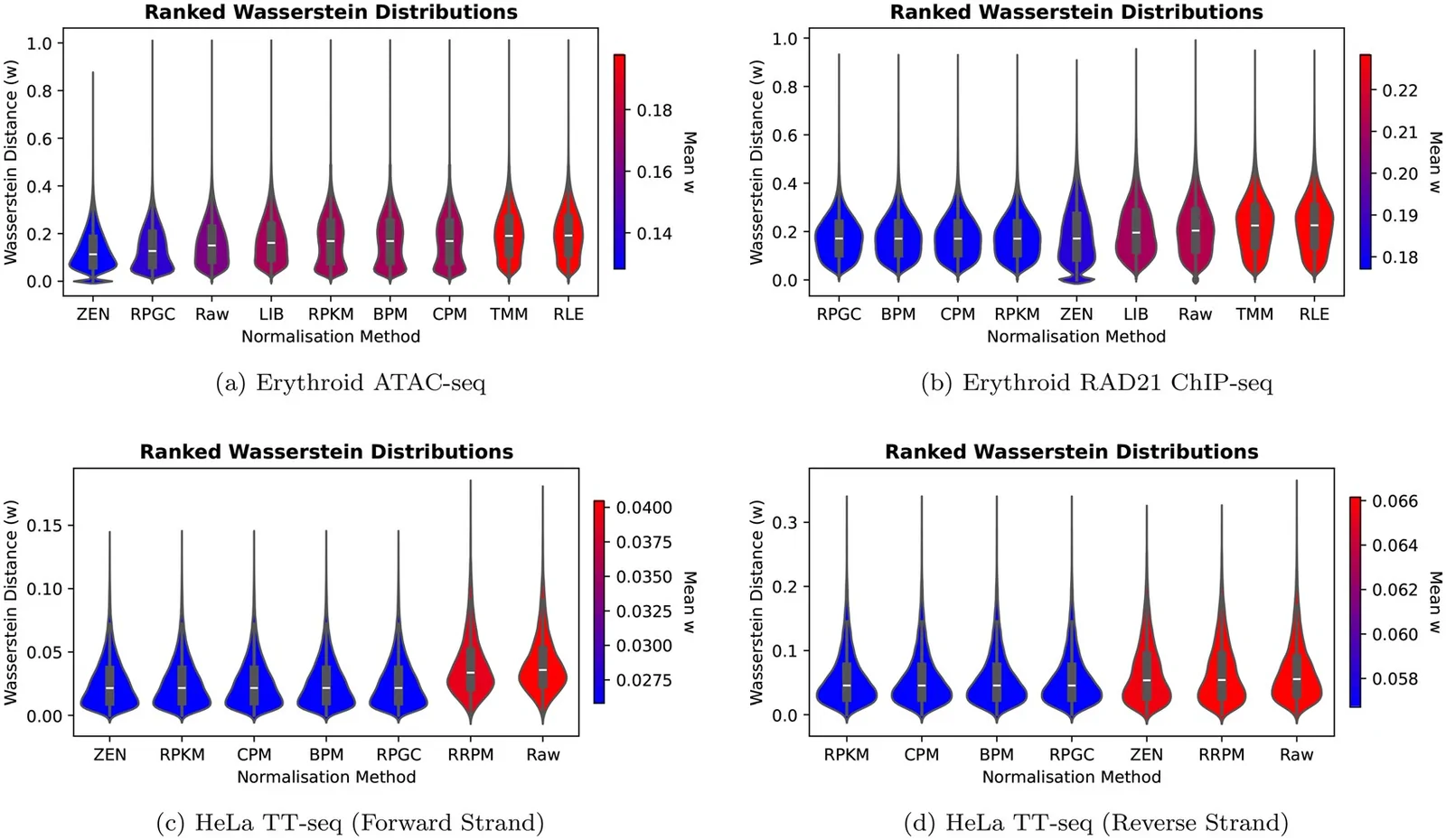
Comparing normalisation methods by min-max scaled Wasserstein distance between sample pairs across signal regions genome-wide. Erythroid ATAC-seq (seven technical reps each for donor 2 and 3) is shown in (a). RAD21 ChIP-seq (three technical replicates for donors 1 and 2, two technical replicates for donor 30) is in (b). Four HeLa TT-seq control technical replicates split into forward and reverse strands are shown in (c) and (d). For ATAC-seq and ChIP-seq, raw and normalised (RPKM, CPM, BPM, RPGC, LIB, RLE, TMM or ZEN) signal regions are compared over LanceOtron peaks. For TT-seq, raw and normalised (RPKM, CPM, BPM, RPGC, RRPM and ZEN) signal regions are compared over padded zones (broad regions of transcription). Violin plots are ranked and coloured by mean Wasserstein distance, where a lower distance implies better alignment.

While ZEN improves biological and technical replicate alignment over key loci, a comprehensive assessment requires determining whether this reduction in variability holds genome-wide across all enriched signal. To do so, we plotted ranked Wasserstein distance distributions across replicates over regions of signal per dataset. For punctate ATAC-seq and ChIP-seq, LanceOtron peaks were used to capture short signal regions, while for TT-seq we used padded zones per chromosome, i.e. $z_{c}^{\prime \prime }$, ∀c∈C, to capture broader regions of transcriptional signal. These values represent genome-wide averages across many regions, so even small reductions reflect consistent improvements in replicate alignment. Figure 4 and Supplementary Results Fig. S12, available as supplementary data at *Bioinformatics* online show violin plots of Wasserstein distance (*w*) from genomic tracks comparing ZEN against all normalisation strategies (including raw) per dataset. Each violin represents the distribution of *w* across regions, where width indicates frequency and colour indicates mean *w*.

For erythroid ATAC-seq and HEK293T, ZEN achieved the lowest ranking mean *w* in pair-wise comparisons with other methods, indicating improvement in consistent signal alignment between samples across enriched regions and reduced non-biological variability. For erythroid RAD21 ChIP-seq and HeLa TT-seq, ZEN performed similarly to the global library size methods, which outperformed the between sample-methods and RRPM. For TT-seq, performance was strand-specific, with ZEN achieving the lowest mean *w* for the HeLa reverse strand, but not for the forward strand.

Most global library size methods performed similarly within each dataset. As they rescale signal and background indiscriminately rather than using biologically relevant signal, their performance may be impacted in experiments with variable high-signal technical artefacts. An exception was RPGC, which performed well in the erythroid ATAC-seq.

For between-sample methods, LIB performed similarly to global library size methods in erythroid ATAC-seq, but worse for RAD21 ChIP-seq. Despite TMM’s and RLE’s attempts to reduce extreme values by trimming and using medians, respectively, they consistently underperformed, suggesting their assumptions designed for RNA-seq counts may not translate well for ATAC-seq or ChIP-seq normalisation.

In TT-seq, RRPM consistently performed the worst, suggesting that spike-ins do not always reliably reduce technical variability. While reverse strand mean *w* was lower than all other methods, global library size methods still performed better for the forward strand.

Overall, mean *w* varied between assay types, with ATAC-seq and RAD21 ChIP-seq displaying means around 0.1–0.2 and TT-seq lower around 0.04–0.1. This may reflect different inter-sample variability between assay types or a consequence of wider regions being compared for TT-seq.

### 3.3 Improved genome-wide sample alignment translates into more conservative differential accessibility analysis results

To test whether lower mean *w* translates to more robust performance and ability to detect true biological differences in cross-sample analyses, we ran DiffBind DA analysis with LIB, TMM, RLE and ZEN on the erythroid ATAC-seq (Supplementary Results Table S2, available as supplementary data at *Bioinformatics* online). To test susceptibility to false positives, we compared donor 2 technical replicates (1–3 vs 4–6) where no biological differences are expected (Supplementary Results Table S2, available as supplementary data at *Bioinformatics* online). While LIB, TMM and RLE predicted thousands of significant peaks, ZEN found only 14, suggesting that other methods may be more susceptible to spurious differences arising from technical noise, whereas ZEN produces a more conservative set of differential peaks.

Comparing donor 2 and donor 3, ZEN predicted the fewest significant peaks (145), followed by LIB (332), RLE (1049), and TMM (1071), consistent with the relative performance of the Wasserstein distance distributions. Inspecting significant peaks, ZEN retained a strong subset of differences in which reduced technical variability improved discrimination between likely true biological differences and false positives. These include increased accessibility over the gene body of *TMEM191B* (Supplementary Results Fig. S13, available as supplementary data at *Bioinformatics* online), suggesting variation in its regulatory landscape in healthy erythroid cells, despite an undetermined functional role in erythropoiesis. ZEN also retained an enhancer downstream of *PPP1R3B*, with single nucleotide polymorphism (SNP) rs3748136 (G$>$A) with gain in function in donor 2, previously associated with increased reticulocyte count (Astle *et al*. 2016) (Supplementary Results Fig. S14, available as supplementary data at *Bioinformatics* online).

Conversely, ZEN excluded many marginal differences likely driven by technical variability, such as a peak in a chromosome 9 non-coding region that was called significant by all methods except ZEN (Supplementary Results Fig. S15, available as supplementary data at *Bioinformatics* online).

## 4 Discussion

Our results show that normalisation choice affects cross-sample signal alignment and downstream analysis outcomes. We introduced ZEN, a novel method that equalises variance across genomic regions. Across diverse genomic datasets (ATAC-seq, ChIP-seq, and TT-seq), we demonstrated that ZEN is an effective and adaptive approach to reduce inter-sample variability and preserve biologically meaningful signal in these datasets over conventional normalisation methods, including global library size based methods (RPKM, CPM, BPM, RPGC), between-sample methods (LIB, TMM, RLE) and spike-in (RRPM). Performance was evaluated over biologically relevant loci, genome-wide Wasserstein distances across peaks or regions of transcription and DA analysis, including technical replicate comparisons, where no biological differences are expected, and biological replicate comparisons to assess detection of robust biological differences.

By correcting technical artefact biases, ZEN reduces cross-sample variability across technical replicates, where no biological differences are expected. This improves signal-to-noise ratios relative to global library size based methods and yields more consistent replicate alignment than RRPM spike-in normalisation—the current standard for TT-seq. Compared to between-sample methods often used for ATAC-seq and ChIP-seq count-based normalisation, reduced technical variability was associated with more conservative differential accessibility results, including fewer calls between technical replicates and retention of biologically plausible differences, such as an enhancer gain-in-function associated with SNP rs3748136 and variable accessibility over the *TMEM191B* gene body, a gene largely uncharacterised in erythroid cells. Even small differences in alignment metrics can lead to qualitatively different downstream results. Although some fundamental differences exist between discrete count matrix and continuous genomic signal normalisation, this suggests that observing signal tracks normalised in the same way counts are scaled can be beneficial.

Together, this shows that normalisation can influence downstream interpretation. Inappropriate normalisation can lead to spurious conclusions, obscure biological signals or introduce artefacts that compromise research integrity. ZEN offers a complementary way to address these challenges via a robust and generalisable genomic data preprocessing framework—an increasingly critical need as sequencing technologies advance and genomic assays grow in complexity.

To extend ZEN’s reach, we developed a reverse normalisation tool, detailed in Supplementary section C, to allow users to work with bigWig files that have been pre-processed by conventional normalisation methods. By reversing existing linear transformations, such as those in bigWigs downloaded from public repositories, analyses can happen that would otherwise be confounded by incompatible normalisation.

Despite the demonstrated advantages of ZEN across most of our diverse set of data, no single normalisation method is optimal for all experimental designs and assays. We therefore advise researchers to evaluate normalisation method suitability and performance based on their own data and research goals. For example, spike-in normalisation would be more appropriate than methods designed to optimise cross-sample alignment in a context with genome-wide signal depletion. Although ZEN was designed to perform well across a wide range of data without parameter tuning, we showed that parameter optimisation can improve context-specific performance.

In summary, ZEN provides a complementary method for researchers to uncover more reliable insights into the molecular mechanisms underlying biological systems. By improving signal alignment and its quantification, as well as reducing technical variability, the genomic research landscape, from individual laboratory studies to large-scale meta-analyses or training ML models, can be improved. Ultimately, this advances our collective understanding of genomic function and regulation by improving the stability and interpretability of downstream analyses.

## Supplementary Material

btag622_Supplementary_Data

## Data Availability

All data used in this study are publicly available. The relevant data sources, links, and identifiers are provided in Section 2.5.
