# Supplementary material for: Zone equalisation normalisation for improved alignment of epigenetic signal
Source: Bioinformatics. 2026 Aug 20;42(9):btag622. doi: 10.1093/bioinformatics/btag622 (PMC13537529; doi:10.1093/bioinformatics/btag622)
Supplement: Supplementary file 1 [file btag622_supplementary_data.pdf]

Supplementary Materials and Methods

A. Additional data

To ensure ZEN is designed to generalise across assay types, we tuned parameters using additional ATAC-seq, ChIP-seq and TT-seq datasets. These include ATAC-seq of brain differentiation from the H1 cell line to forebrain, midbrain and hindbrain, with three replicates per stage (GSE286146), and single cell ATAC-seq (scATAC-seq) bigWigs of 111 human cell types CATlas portal (Zhang et al., 2021). Erythroid CTCF ChIP-seq from Donors 1, 2, and 30 bigWigs were downloaded from the same study as the RAD21 ChIP-seq (GSE244929) (Georgiades et al., 2025). RNA Polymerase II Ser2P (Pol II) ChIP-seq was included from the HEL leukaemia cell line with three replicates (GSE84157). TT-seq was obtained of the A-375 melanoma cell line comparing a CDK13 mutant clone R860Q with a control (GSE223888) (Insco et al., 2023).

All raw data was aligned using the CATCH-UP pipeline with default parameters without RPKM normalisation. A-375 TT-seq was mapped to a combined hg38 and dm6 genome created with SeqNado. For the MACS2 Signal Per Million Reads (SPMR) (Zhang et al., 2008) normalised CATlas bigWigs and RPKM normalised erythroid CTCF ChIP-seq bigWigs, pre-normalisation was reversed to create raw bigWigs using the method described in Supplementary section C.

B. ZEN normalisation workflow

B.1. Distribution fitting

Due to low signal-to-noise ratios within genomic signal for most assays, histograms of smoothed signal $\tilde{y}$ are typically skewed. Removing zeros and log-transforming $\tilde{y}$ creates a more symmetrical signal y'_c, which is more suitable for parametric distribution fitting. For example, Figure S1 compares the shape of the $\tilde{y}_c$ and y'_c histograms for an erythroid ATAC-seq replicate.

To determine the best default distribution to generalise across assays, distributions with two parameters were fitted per $c \in \mathcal{C}$ for each sample in each ATAC-seq, ChIP-seq and TT-seq dataset. These parameters are the location L_c (the shift of the distribution) and scale V_c (the statistical dispersion). The set $\mathcal{D}$ of distributions includes: Normal, Logistic, Laplace, Gumbel (left or right) and Cauchy.

For each distribution $d \in \mathcal{D}$, three types of (L_c, V_c) parameters (referred to as parameter type) were fitted. The first infers (L_c, V_c) by maximum likelihood for d using SciPy (Virtanen et al., 2020). The second sets $L_c = \tilde{M}'_c$ and the third sets $L_c = \tilde{\mu}'_c$, where $\tilde{M}'_c$ is the median and $\tilde{\mu}'_c$ the mean of $\tilde{y}'_c$ retrospectively. For these, scale V_c is determined by Eq. 1 using constants specified in Table S1.

$$V_c = \begin{cases} \alpha_d \cdot \mathrm{MAD}(\tilde{y}'_c), & L_c = \tilde{M}'_c \wedge \mathrm{MAD}(\tilde{y}'_c) \neq 0 \\ \beta_d \cdot \mathrm{MeanAD}(\tilde{y}'_c), & L_c = \tilde{M}'_c \wedge \mathrm{MAD}(\tilde{y}'_c) = 0 \\ \gamma_d \cdot \tilde{\sigma}'_c, & L_c = \tilde{\mu}'_c \end{cases} \tag{1}$$

	**Scale Constants**		
d	α_d	β_d	γ_d
Normal	1.4826	1.2533	1
Laplace	1.4427	1	0.7071
Logistic	1	0.7213	0.5513
Cauchy	1	1	1
Gumbel	1.3036	1.6654	0.7797

Table S1. Constants for deriving the scale of a distribution d. α_d and β_d are used if d is centred using the median and γ_d if centred using the mean. Each d sets dispersion differently, so α_d is set using the distribution's definition of MAD, β_d from its MeanAD and γ_d from its SD with several exceptions: Cauchy distribution variance is undefined, so α_d, β_d and γ_d are set as one; Logistic distribution MAD is undefined, so $alpha_d$ is one; Gumbel distribution α_d was derived from numerical approximation by A. Akinshin (Akinshin, 2020; Heckert et al., 2002; Harrell and Davis, 1982).

Quantile-Quantile (Q-Q) plots were created to compare shape agreement between the empirical distribution of a sample's transformed smooth signal y'_c per chromosome with candidate theoretical distributions. Empirical quantiles of y'_c were therefore plotted against corresponding theoretical quantiles for each fitted distribution. Figure S2 shows Q-Q plots for chromosome 9 of three samples across experiment types. Although distributions were fit with the median parameters, Q-Q plots assess distributional shape independently of location and scale. Therefore the closer the points align with the red line, the better the agreement of the distribution's shape regardless of its parameters. For ATAC-seq, ChIP-seq and TT-seq samples, y'_c shows lighter tails than expected under the Cauchy distribution and Cauchy was therefore excluded from further analyses.

To further investigate distribution shape and parameter fit, histograms of y'_c were created per chromosome. Figure S3 shows how distribution fitting varies with different parameters for erythroid ATAC-seq and HeLa TT-seq. As genomic assays are often noise-heavy, most histogram bins represent background noise. Across all ATAC-seq and ChIP-seq datasets, histograms were unimodal

and relatively symmetrical. Therefore, Gumbel distributions were not considered appropriate for these assays. Most others provided reasonable fits, though Normal did not always match the empirical data when using the mean or SciPy estimated parameters.

By contrast, the HeLa and HEK23T TT-seq exhibited skewed and weakly bimodal distributions. This may reflect differing noise distributions between punctate assays and those with broader regions of genomic signal. The rightmost mode may represent weak but widespread transcriptional activity. However, this was not universal as A-375 TT-seq was more symmetrical suggesting dataset-specific factors may also contribute.

Additionally, Figure S3 shows how a fixed zone probability p translates to a different PPF(p), and consequently zone threshold λ_c depending on the distribution's tail behaviour. For example, the default $p = 0.995$ for Laplace produces a slightly higher λ_c than Logistic, as heavier-tailed distributions have higher probability in the extremes.

To quantify the performance of each distribution and parameter type per assay, the mean Kolmogorov-Smirnov (KS) statistic, D_δ, was calculated for each chromosome per sample across multiple datasets. The mean D_δ for each dataset per assay are shown as box plots in Figure S4. For ATAC-seq, the distribution with the lowest average D_δ was Laplace with median parameters. For ChIP-seq, the lowest were Laplace and Logistic with SciPy parameters. While for TT-seq, Logistic with SciPy parameters fitted best. As both Laplace and Logistic generally showed comparable performance, we selected the Laplace distribution with median parameters as the default due to the median's robustness to outliers.

B.2. Signal zone prediction and quality filtering

During zone prediction, thresholding the smoothed signal by a minimum of r consecutive bp aims to select biologically plausible initial signal regions while removing most noise. Most transcription factor (TF) motifs are between 5-20 bp (Aptekmann et al., 2022). However, short read sequencing technologies keep fragments of at least 50 bp (Goodwin et al., 2017), while the typical ATAC-seq and ChIP-seq fragment size is between ∼100-600 bp (Diagenode; Yan et al., 2020). Consequently, enriched peaks often span at least 100 bp.

The default value $r = 35$ was chosen to capture regions that are longer than individual motifs yet shorter than full peak widths. This ensures that detected regions likely represent meaningful biological signals, including motifs and their immediate context, while allowing for minor local signal fluctuations within broader peaks. Although signal is smoothed by a kernel, the default κ_{301} was designed to reduce the influence of isolated short spikes of signal, but still largely preserves the structure of peaks in their original positions.

For example, Figure S5 shows unnormalised ATAC-seq from erythroid donor 3 (technical replicate 6) over the functionally important *NPRL3* locus. This demonstrates how open chromatin peaks correspond with regions enriched in short motifs, such as *KLF4*, *NFX1*, *SP4* and *CTCF*. The zone threshold line λ_c is the cut-off by which the signal must consecutively exceed for 35 bp or more to be classed as signal over noise prior to quality filtering. This shows how enriched signal is successfully separated from background noise.

After thresholding by λ_c, some low quality signal can remain. As λ_c is chromosome-specific, it may not exclude all noise if predicted for a very sparsely mapped chromosome, such as chromosome Y. Technical artefacts, such as PCR duplicates or mappability issues, can also be problematic by causing high isolated values. Given most assays are vulnerable to these, applying a second threshold, quality filter τ_c, to remove regions with less than $m = 5$ bp aims to reduce the amount of low-quality signal included in zones.

As τ_c is based on local and global features of the signal per sample, it automatically adapts to the data. For sparse data, the non-zero means are low and so τ_c evaluates to $10\hat{f}_c$. This is similar to filtering bins with less than 10 reads, as is often performed during DB analysis to ensure signal is sufficient quality (Love et al., 2014; Lun and Smyth, 2015). For more deeply sequenced data, 10 reads may not be sufficient so τ_c evaluates to either 1.5× the chromosome-specific or genome-wide mean (excluding zeros). A scale of 1.5 was selected as it approximates the upper limit for most values within noise.

For example, Figure S7 shows low-quality, unnormalised chromosome Y signal in erythroid donor 1 RAD21 ChIP-seq technical replicates 1-3. Even though replicate 1 exceeds τ_c by over 200 bp, the signal is blocky and only has three consecutively different values exceeding τ_c. With $m = 5$, this region is excluded from predicted zones.

B.3. ZEN parameter exploration

After setting the default parameters as outlined in Methods and Materials, we explored how changing user-adjustable parameters affects replicate alignment in erythroid ATAC-seq, erythroid RAD21 ChIP-seq, HeLa TT-seq and HEK293T TT-seq. To assess the impact of smoothing strength, we tested triangular smoothing kernels κ_n with size $101 \leq n \leq 501$. For distribution fitting, we compared fitting to the full transformed smooth signal $\tilde{y}'_c$ with down-sampling to $100 \leq \delta \leq 1,000$ values. After fitting a Laplace distribution, we evaluated how zone prediction was impacted by adjusting zone probability as $0.9 \leq p \leq 0.999$, minimum region size as $10 \leq r \leq 100$ and minimum region difference as $1 \leq m \leq 100$. We also assessed the impact of normalising from padded zones with padding bin size $100 \leq b \leq 2,000$ or from unpadded zones (no binning). Benchmarking performance (Figure S6) was quantified by mean Wasserstein distance w across LanceOtron peaks (ATAC-seq and RAD21 ChIP-seq) or padded zones predicted

with the default parameters (TT-seq).

The default kernel size, $n = 301$, achieved a good baseline performance. For the punctate assays, $n \leq 101$ slightly decreased performance, while $301 \leq n \leq 501$ had minimal effect. This implies that smoothing improves the proportion of zones capturing signal rather than background noise without substantially distorting peak structure.

For distribution fitting, $\delta = 300$ produced comparable mean w values to fitting without down-sampling. This suggests that fitting the distribution to a sub-set of signal $\tilde{y}'_c$ does not impair performance. Although down-sampling is seeded for reproducibility, it could introduce some variability and therefore may be disabled if this is of concern. Zone probability had one of the largest effects across assays, with a lower p showing better performance in all datasets suggesting alignment can be improved with a stricter cut-off between signal and background when predicting zones.

For signal zone prediction, a minimum region size of $r = 35$ performed well, though adjusting it made little impact for most datasets. In contrast, minimum region difference size had a greater effect. Performance improved in the erythroid ATAC-seq for $m > 5$, but reduced for RAD21 ChIP-seq with $m > 20$. In theory, increasing r and m improves the quality of signal predicted in zones, but at the cost of zone signal retention.

Padding bin size correlated with performance for most assays, with $b < 500$ beneficial for erythroid ATAC-seq and HEK293T TT-seq, whereas $b > 1000$ was marginally better for the RAD21 ChIP-seq. Larger b values add padding to zones which includes background signal. Therefore, reducing b likely has a similar effect on the calculated normalisation scaling factors as increasing p or m through reducing the influence of background signal.

Overall, this shows that the default parameters provide a stable baseline, but that dataset and assay specific optimisation can improve performance further. For example, although $p = 0.995$ performed well for the tested assays, assays with broad chromatin enrichment (e.g. H3K36me3 or H3K27me3) may contain signal across more than 0.5% of the genome. Therefore a reduced p could be tested to lower the stringency of the signal–background cutoff for zone prediction. Moreover, we demonstrated adjusting one parameter at a time to test their effect, but performance could be optimised further by adjusting multiple parameters simultaneously.

$$W_{ijk} = \beta_0 + \beta_1 N_k + u_j + \epsilon_{ijk} \tag{2}$$

An LMM was selected as distributions consist of non-independent measures of similarity over genomic regions across replicates. As p-values are not from independent tests, false discovery rate (FDR) was controlled for by adjusting p-values by Benjamini-Yekutieli (BY) (Benjamini and Yekutieli, 2001).

C. Reverse normalisation

For an unnormalised signal $y = y_1, \dots y_N$, where N is the genome size, a linearly normalised signal is derived by multiplying with a scaling factor $s \in \mathbb{R}^+$ (Eq. 3). Examples of such linear normalisation methods include RPKM, CPM, BPM, RPGC, LIB, TMM, RLE, RRPM and ZEN.

$$y^\eta = y \cdot s \tag{3}$$

Linear normalisation can therefore be reversed using the reciprocal of s. But in practice, it can be difficult to recover y if s is unknown. For example, with RPKM normalisation, s relates to the library size (the total reads for the sample), and without access to the raw data, such as BAMs, s cannot be determined. However, given the high abundance of background noise in sequencing data, it is often possible to reconstruct y with high accuracy by estimating s.

Scaling factor estimation relies on the principle that the fragment size f, representing the contribution of a single read in y, is +1 if the signal is all positive and −1 otherwise. After linear normalisation, the per-read contribution of ±1 is scaled. Therefore, the normalised fragment size f^η becomes $\pm s$, and s is the absolute value of f^η (Eq. 4).

$$s = |f^\eta| \tag{4}$$

For most genomic assays, the abundance of background noise makes it highly likely that there exists at least one i such that $y_i^\eta = f^\eta$. The normalised fragment size $\hat{f}^\eta$ can therefore be estimated as the smallest non-zero value genome-wide (Eq. 5).

$$\hat{f}^\eta = min(\{y_i^\eta \; | y_i^\eta \neq 0, 1 < i < N\}) \tag{5}$$

Consequently, the estimated scaling factor $\hat{s}$ is defined as the absolute value of this estimation (Eq. 6).

$$\hat{s} = |\hat{f}^\eta| \tag{6}$$

If $\hat{s} = 1$, this implies either $y^\eta = y$ or the normalisation was non-linear. To distinguish between these cases, the signal is checked to test whether $\forall i \in [1, \cdots N] : y_i^\eta \in \mathbb{Z}$. If this holds, then the signal has likely not been normalised. Otherwise, a normalisation may have been applied that cannot be reversed by division with a constant. If $\hat{s} \neq 1$, it is assumed that a linear normalisation was applied. In this case, the reciprocal of the sample-specific scaling factor is used to calculate the reversed normalised signal $\hat{y} = \hat{y}_1 \cdots \hat{y}_N$ (Eq. 7).

$$\hat{y} = \frac{y^{\eta}}{\hat{s}} \tag{7}$$

To correct for floating point errors, values in $\hat{y}$ are rounded to the nearest integer and saved to a bigWig file. Assuming $\hat{s}$ was estimated within a small margin of error, the original signal prior to linear normalisation can be accurately reconstructed.

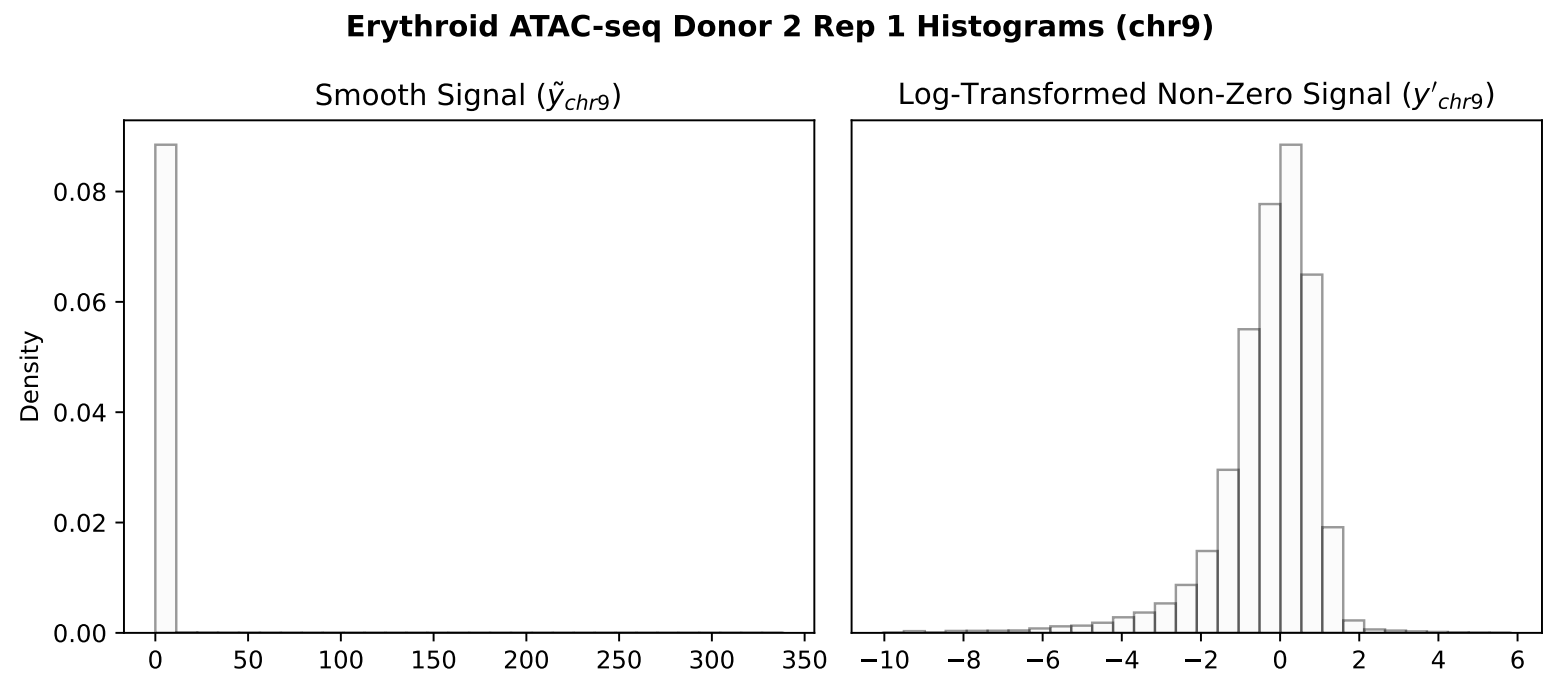

Figure S1. Histograms of raw signal y_c (left) and transformed smooth signal y'_c (right) for erythroid ATAC-seq donor 2 rep 1. The y_c histogram is bias towards near-zero values, while y'_c histogram is less bias.

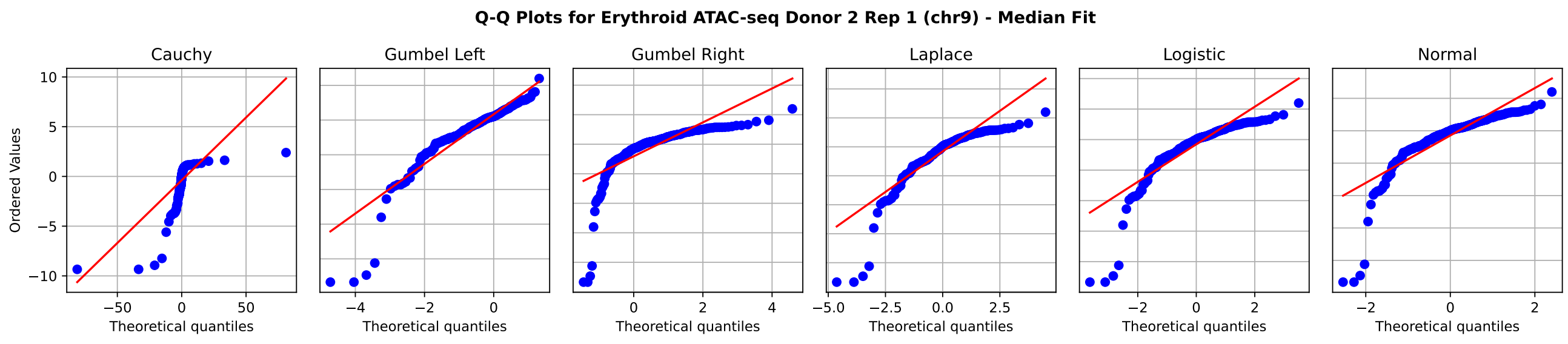

(a) Erythroid ATAC-seq

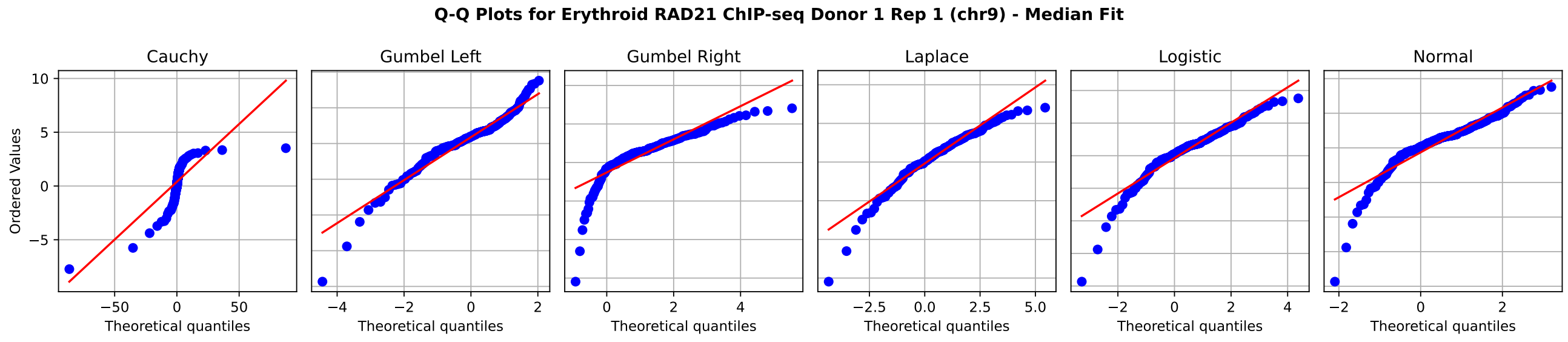

(b) Erythroid RAD21 ChIP-seq

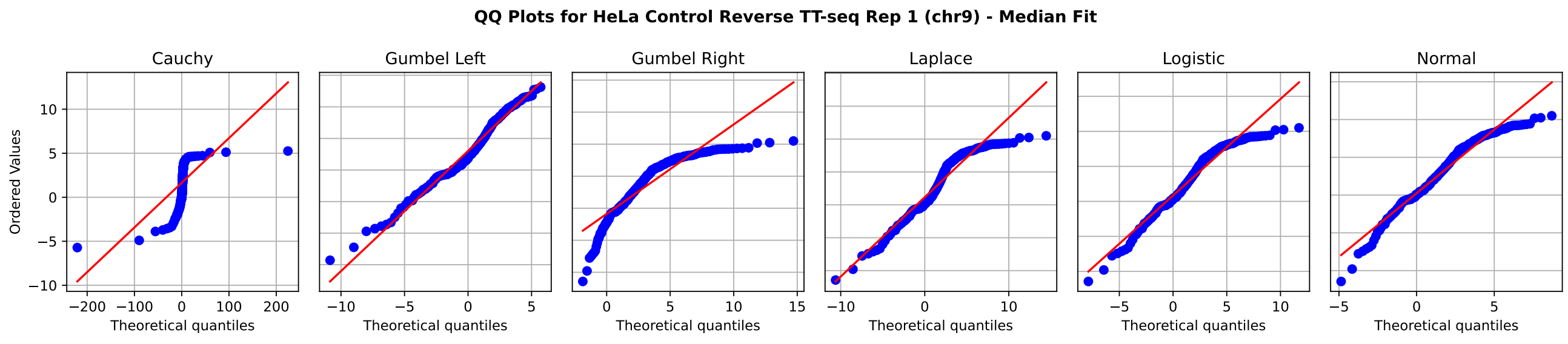

(c) HeLa TT-seq

Figure S2. Q-Q plot per distribution fitted with median parameters to transformed, down-sampled smooth signal $\tilde{y}'_c$, where c is chromosome 9 and $\delta = 300$. Panels show: (a) erythroid Donor 2 ATAC-seq rep 1, (b) RAD21 ChIP-seq rep 1 and (c) Hela TT-seq rep 1. Red lines represent expected values for each theoretical distribution. Blue dots are empirical quantiles from the absolute value of chromosome 9 signal after smoothing, exclusion of zeros and log-transformation.

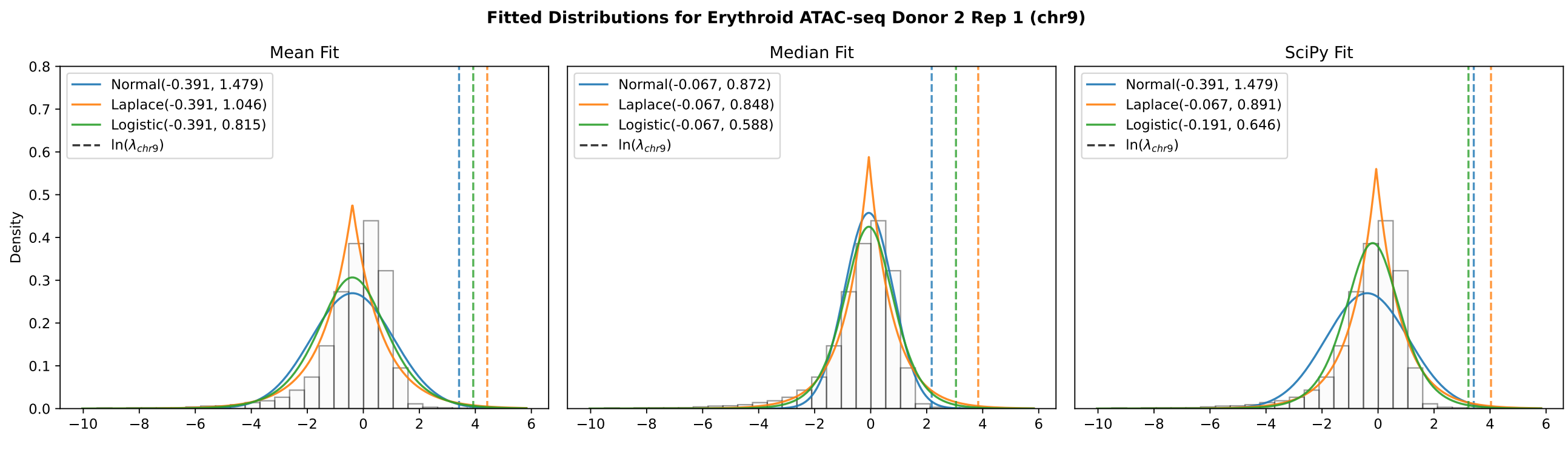

(a) Erythroid ATAC-seq

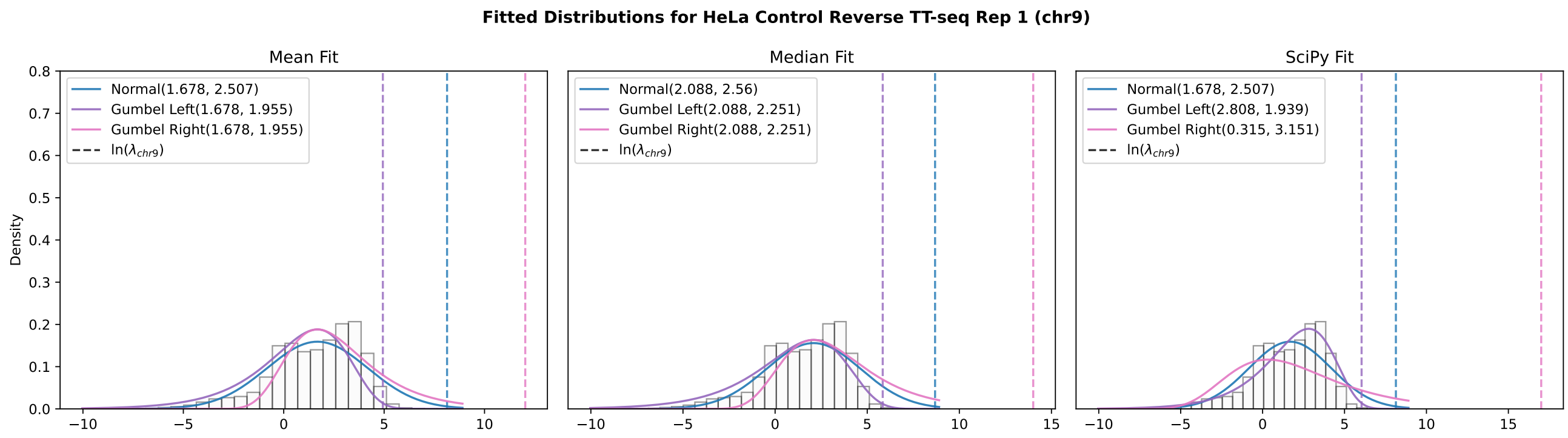

(b) HeLa TT-seq

Figure S3. Histograms of transformed smooth signal y_c' and distributions fitted with the mean (left), median (centre) and SciPy (right) parameters. Normal, Laplace and Logistic distribution are show for erythroid ATAC-seq (a), while Normal, Laplace and Logistic are show for HeLa TT-seq (b), Normal and Gumbel distributions. In the legend, distribution names are given along with their location (L_c) and scale (V_c). Dashed lines represent the CDF's position for 0.995 zone probability. This is equivalent to the log transformed signal-to-noise threshold λ_c.

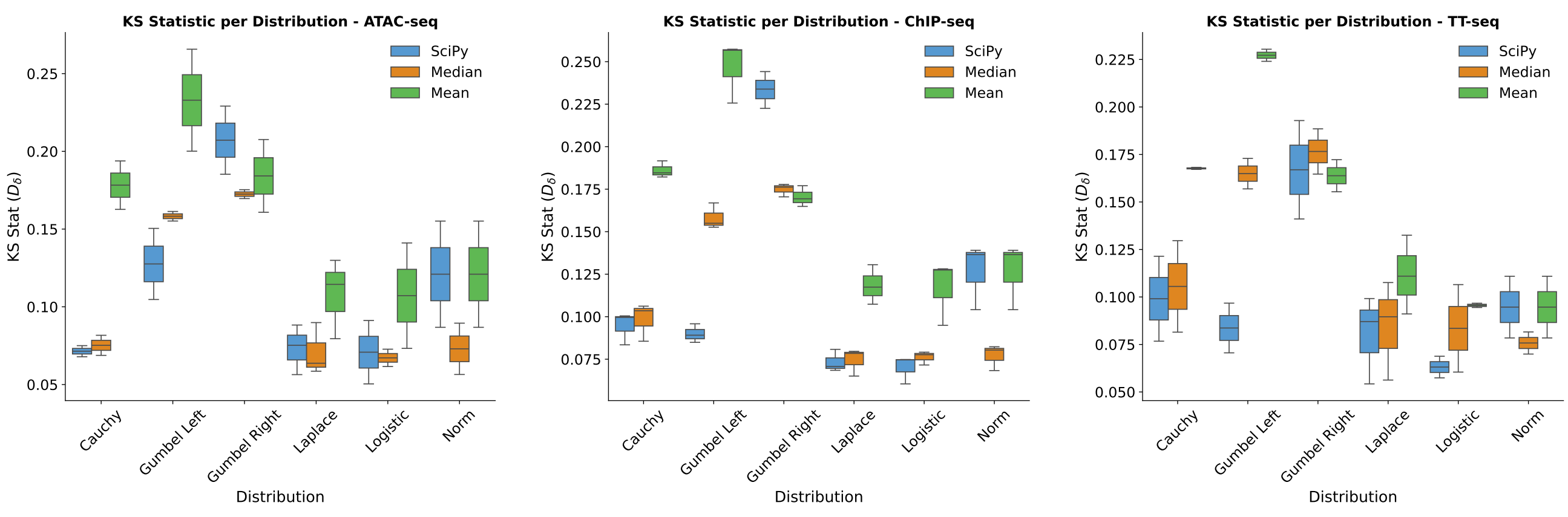

(a) ATAC-seq (b) ChIP-seq (c) TT-seq

Figure S4. KS test results of each distribution and parameter type fitted prior to signal zone prediction. For (a), tested datasets include erythroid Donor 2 and 3 ATAC-seq, H1 brain ATAC-seq and CATlas scATAC-seq. For (b), ChIP-seq datasets include Erythroid RAD21, Erythroid CTCF and HEL Pol II. For (c), TT-seq dataset include HeLa, A-375 CDK13 (mutant and control) and HEK293T.

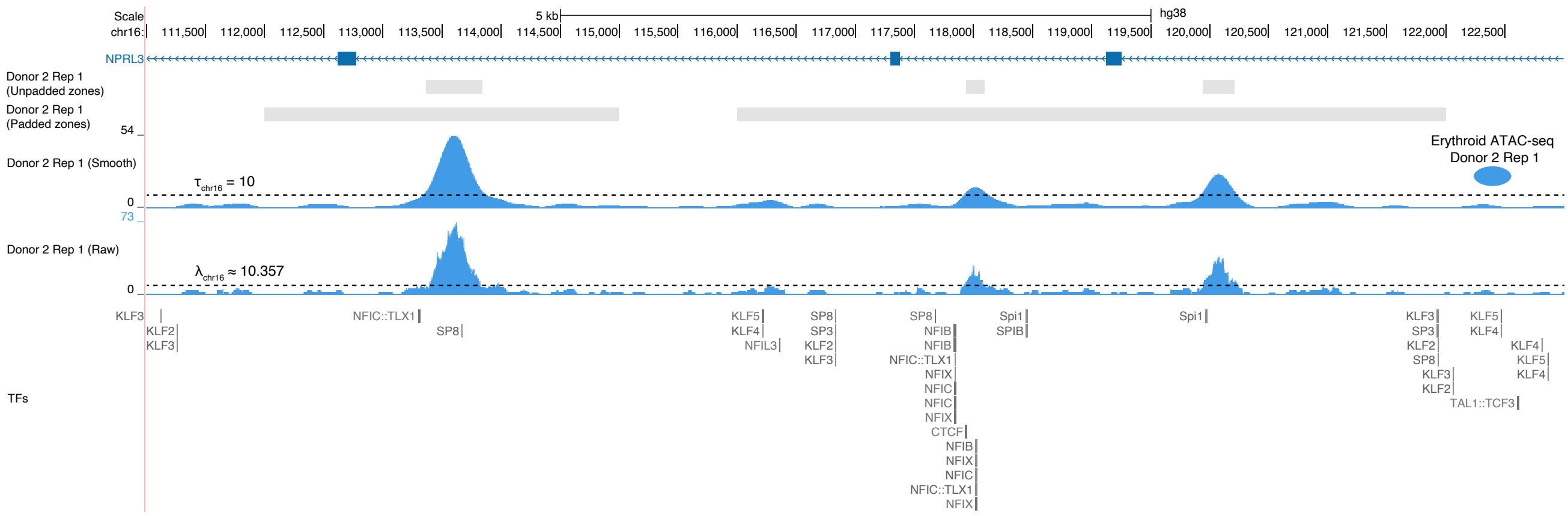

Figure S5. Chromatin accessibility signal tracks (chr16:111,000-123,000) from erythroid donor 3 (rep 1) unnormalised signal before (lower track) and after (top track) smoothing. Three open chromatin peaks are shown, where the left peak is over globin enhancer R2 (chr16:112,529-114,645). Over the smoothed signal, zone threshold $\lambda_{chr16} \approx 10.357$ shows the cut-off which signal must consecutively exceed by r bp to be considered enriched prior to quality filtering. Over the non-smoothed signal, the quality filter threshold $\tau_{chr16} = 10$ shows the value that must be exceeded by m or more consecutive bp differences to be included within zones. Above the tracks, coordinates of zones are shown before (z''_{chr16}) and after padding (z'''_{chr16}). Lower annotations are selected TF binding sites (JASPAR), representing short DNA sequences recognised by specific TFs.

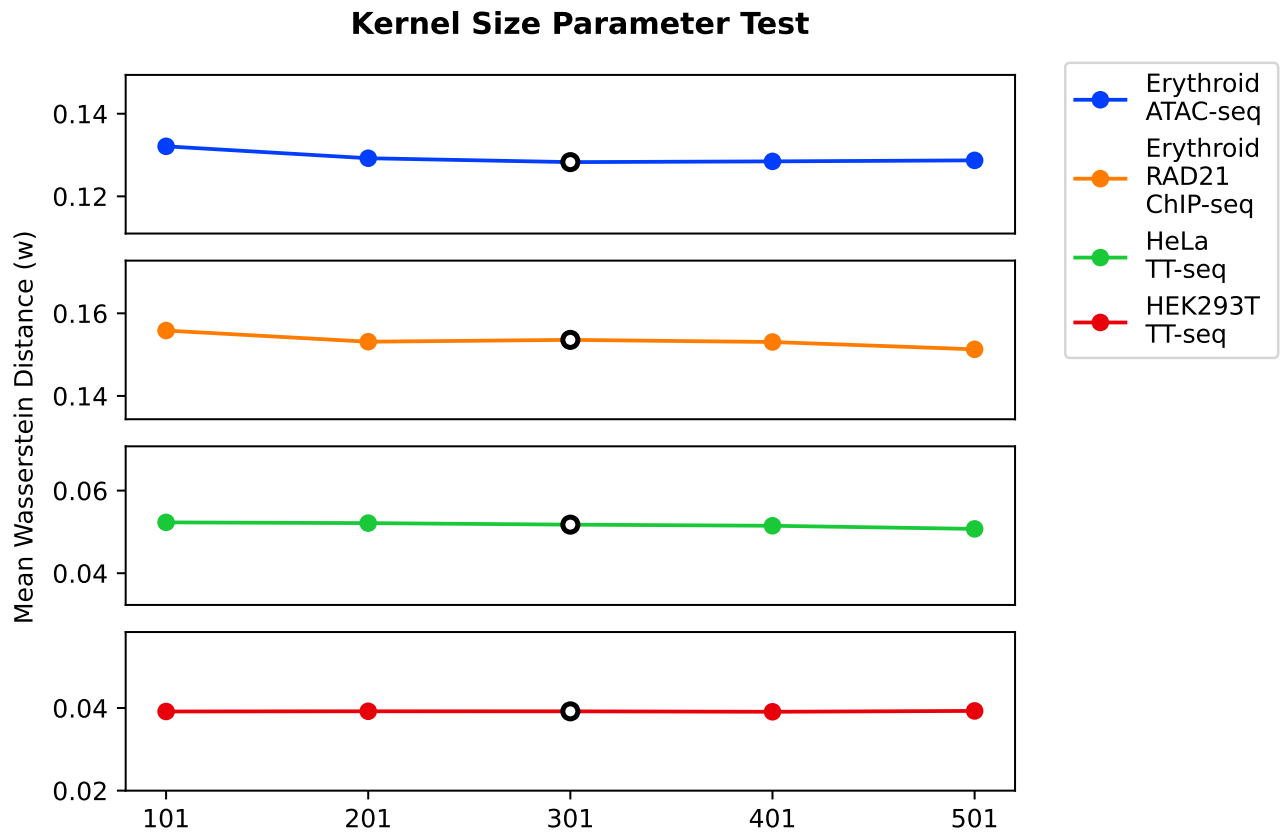

(a) Kernel size (n)

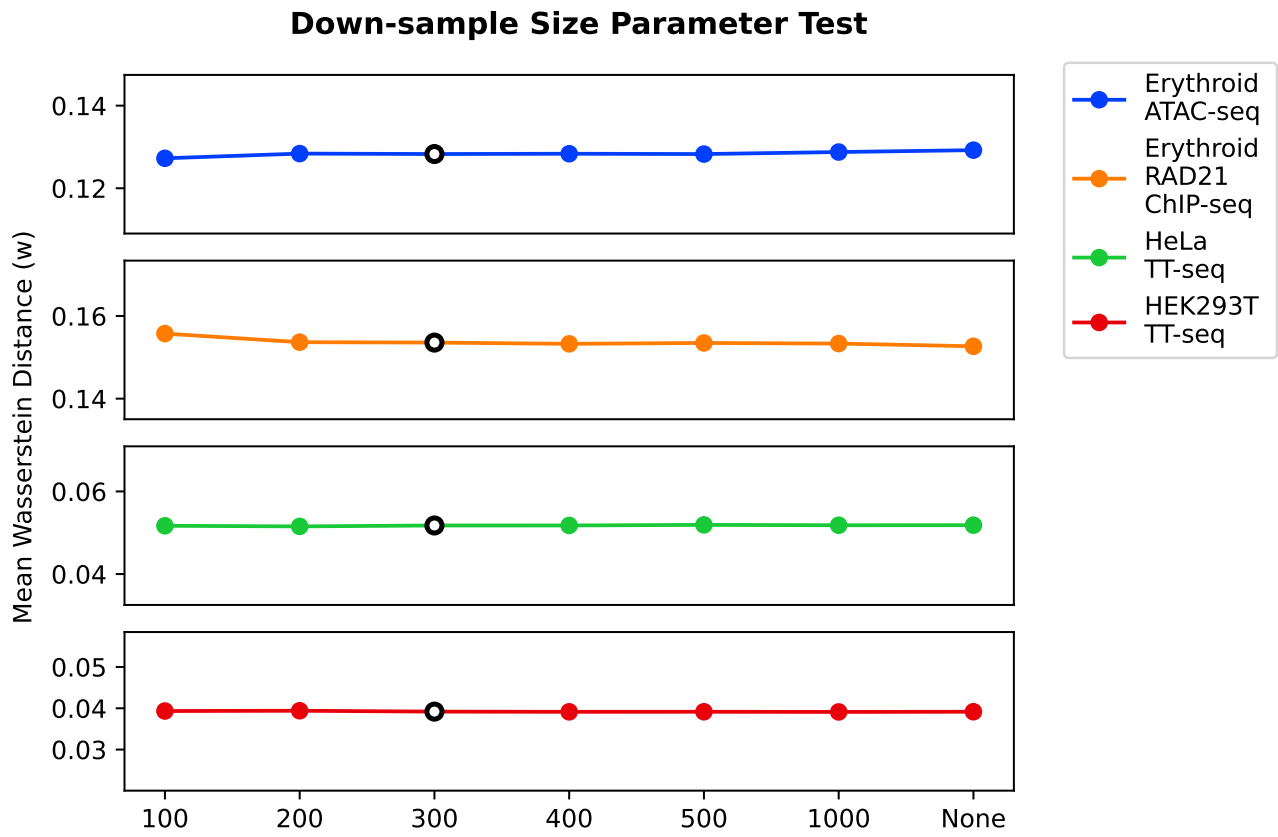

(b) Down-sample size (δ)

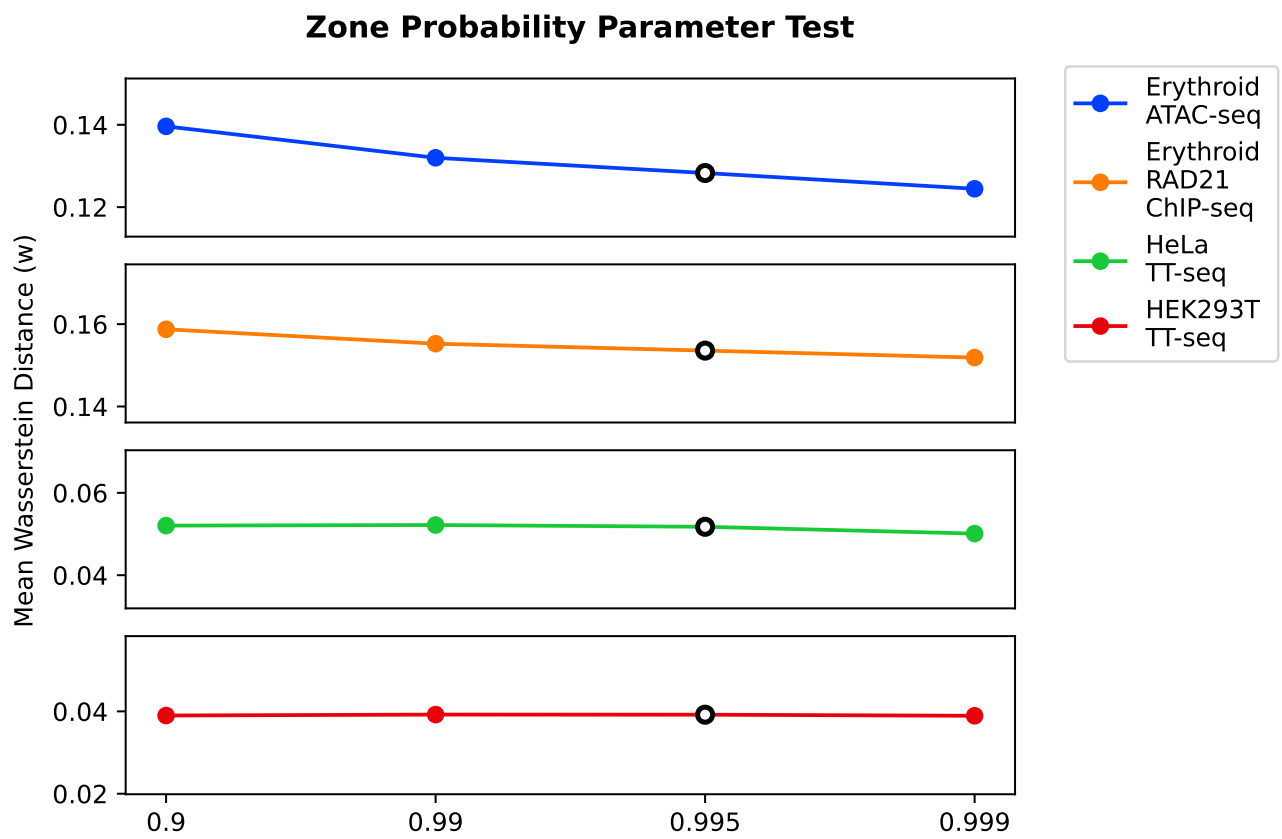

(c) Zone probability (p)

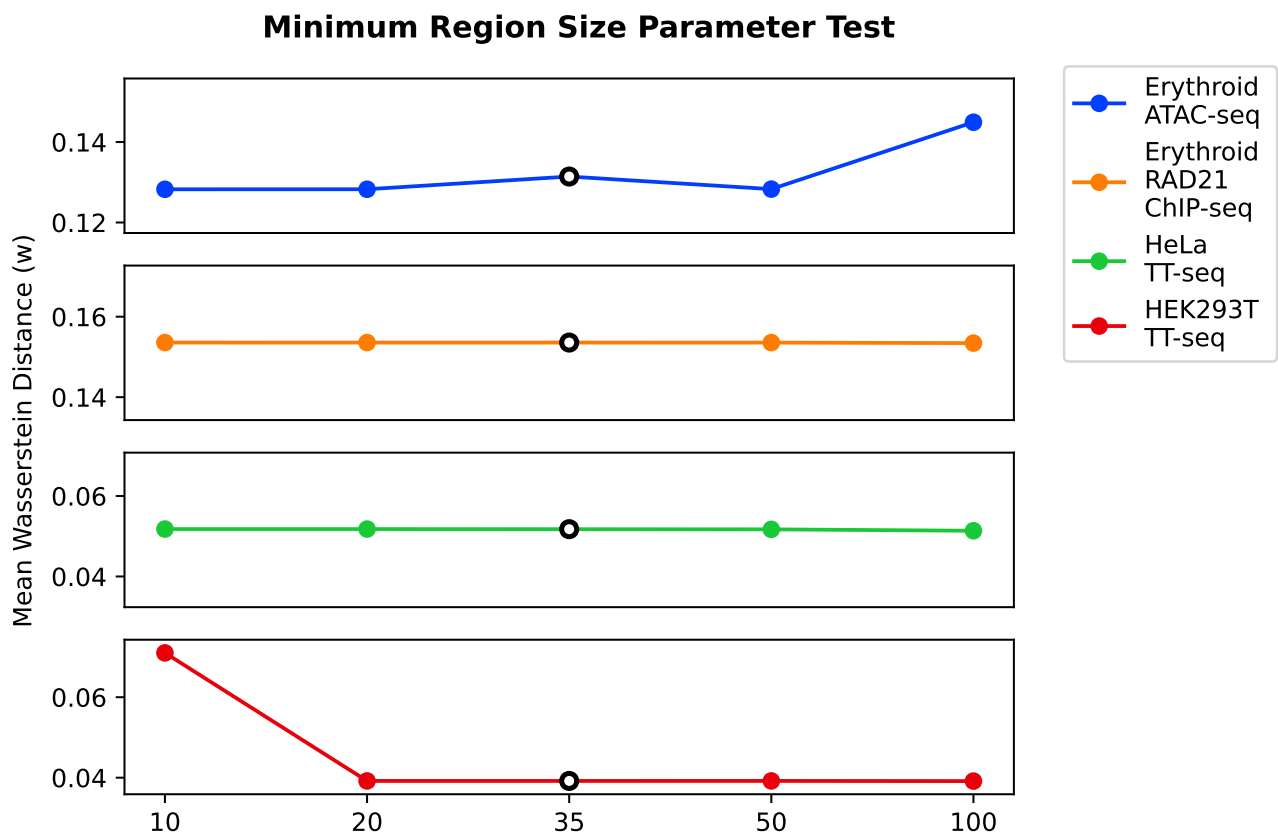

(d) Minimum region size (r)

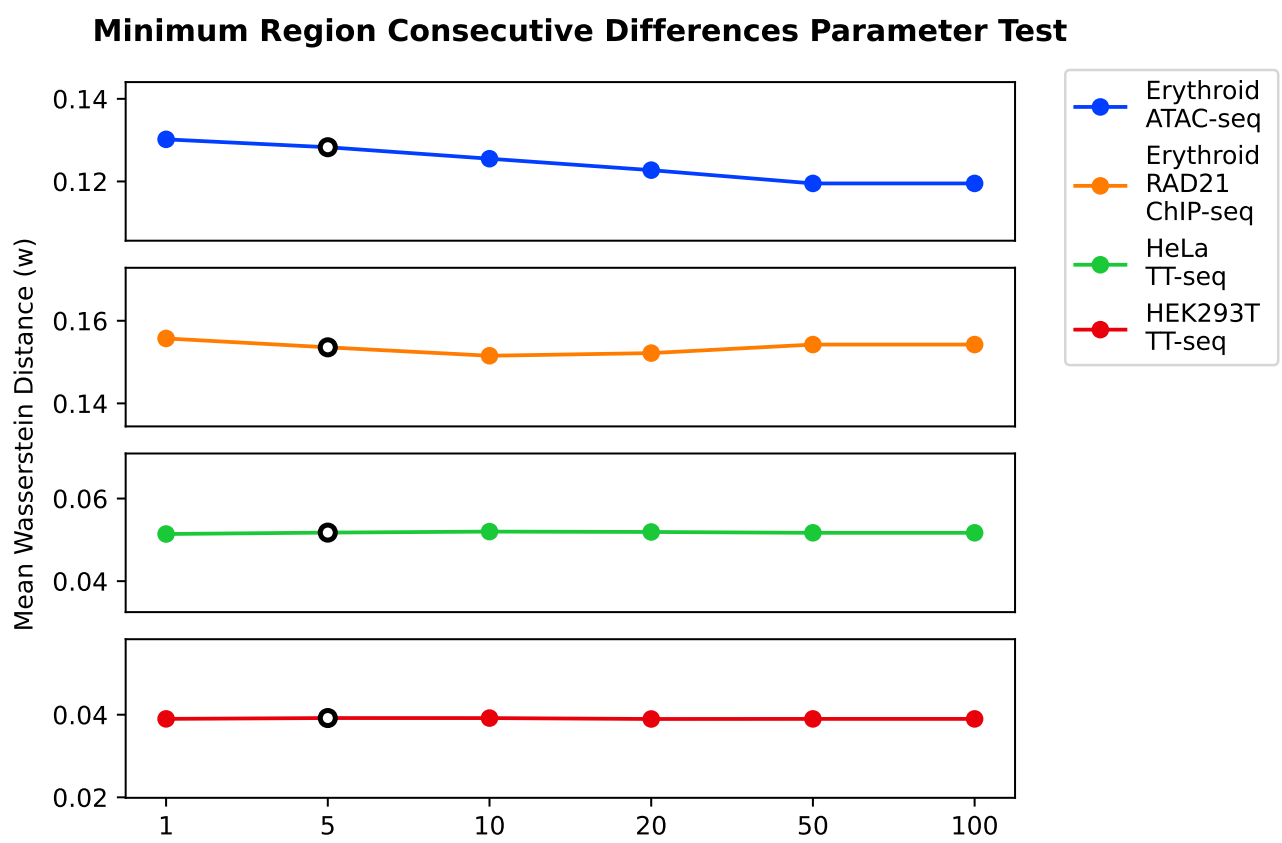

(e) Minimum region difference (m)

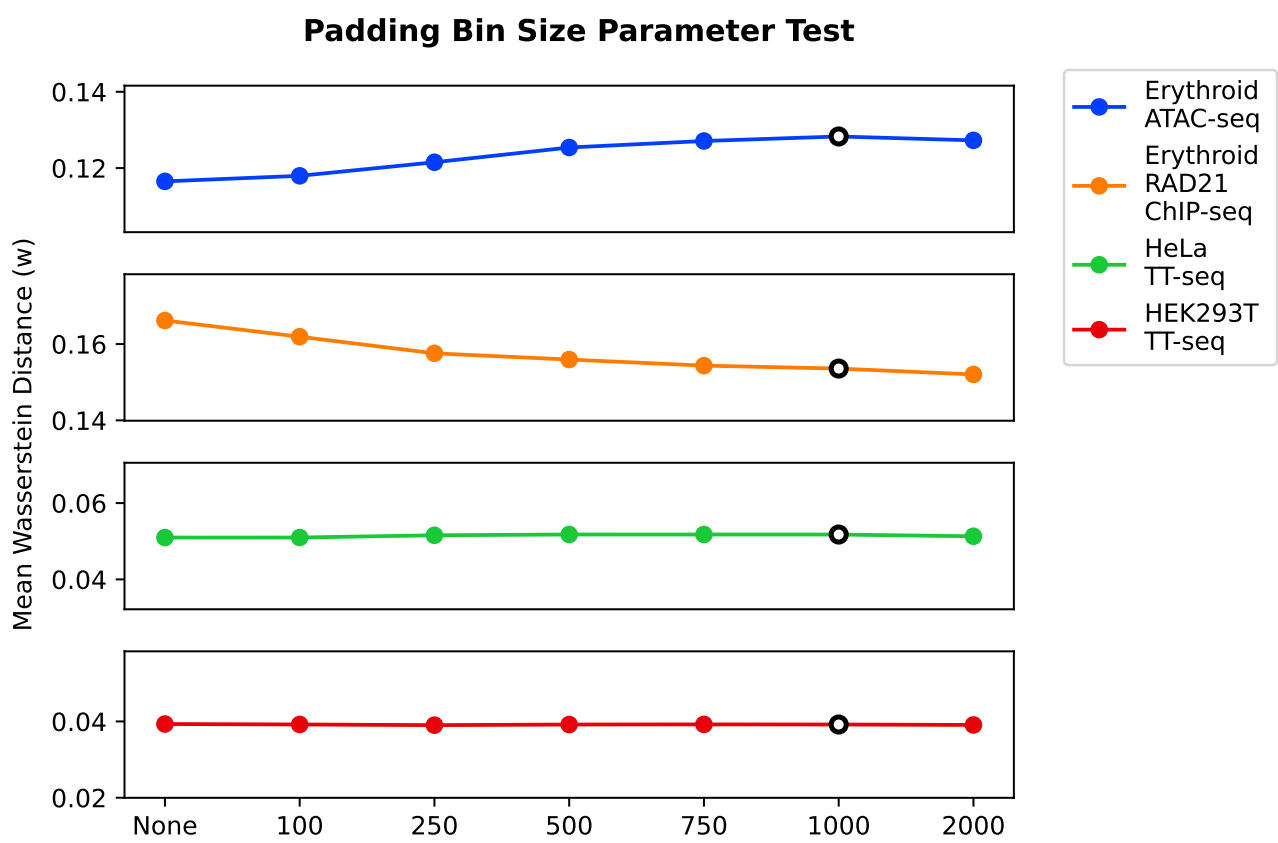

(f) Padding bin size (b)

Figure S6. Plots showing how the performance of ZEN's genome-wide signal alignment across erythroid ATAC-seq (blue), erythroid RAD21 ChIP-seq (orange) and HeLa TT-seq (green) is affected by changing values of parameters triangular kernel κ_n of size n (a), down-sampling size δ (b), zone probability p (c), minimum region size r (d), minimum region difference m (e) and padding bin size b (f) from the default. Performance is measured by mean Wasserstein distance (w) across LanceOtron peaks for ATAC-seq and ChIP-seq and padded zones (broad regions of transcription) for TT-seq. Lower w implies stronger alignment and results with the default parameters are highlighted as black hollow circles.

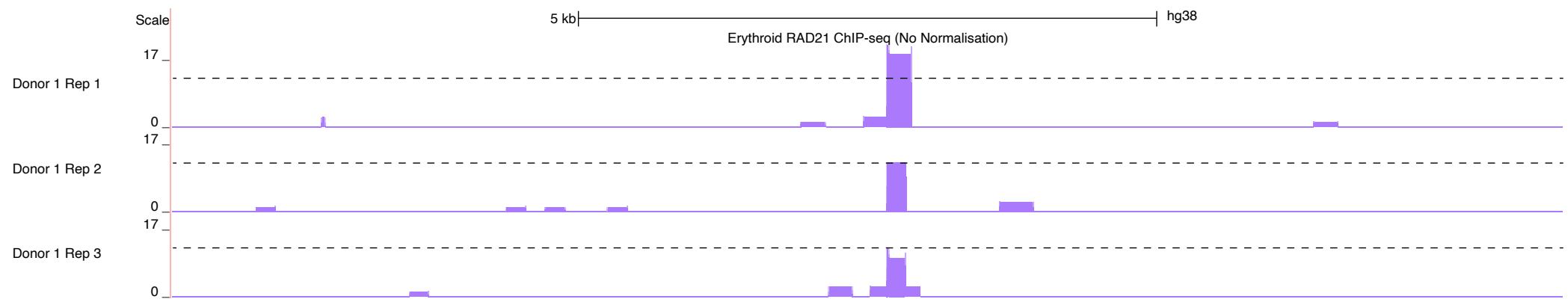

Figure S7. Genome browser view of erythroid RAD21 donor 1 (purple) ChIP-seq coverage over chromosome Y (chrY:8,853,000-8,865,000). Tracks show a region of unnormalised background noise over technical replicates 1-3. The black line represents the quality filter threshold $\tau_{chrY} = 10$. In the centre is a block of low-quality signal that exceeds τ_{chrY} by ~ 200 bp in rep 1, but only has three consecutive different bp values above τ_{chrY}.

Supplementary Results

D. Genome browser tracks

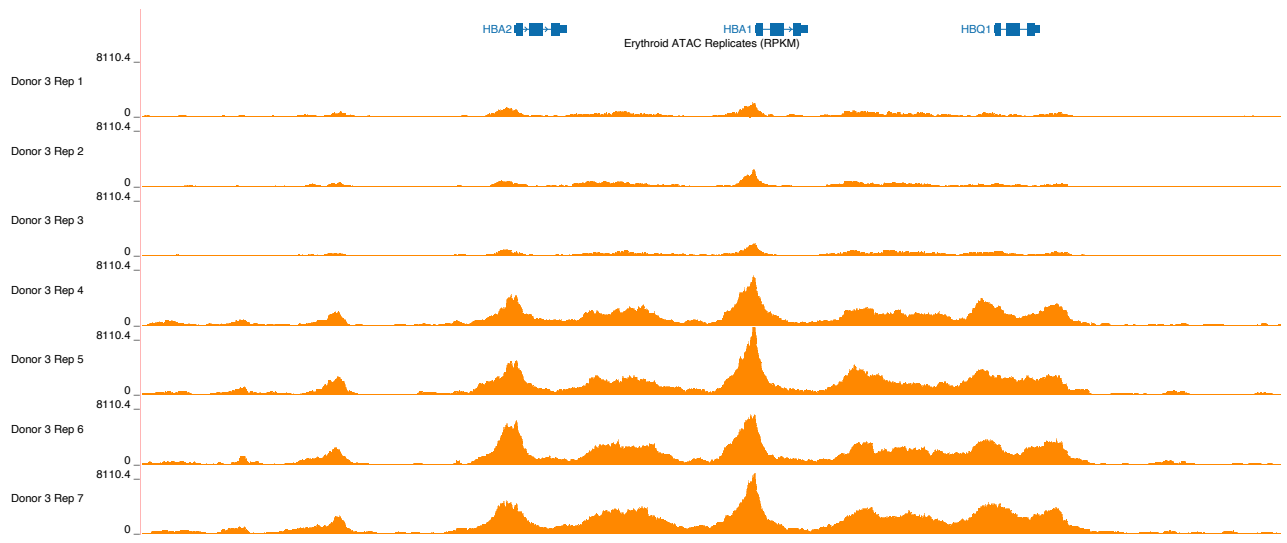

(a) RPKM

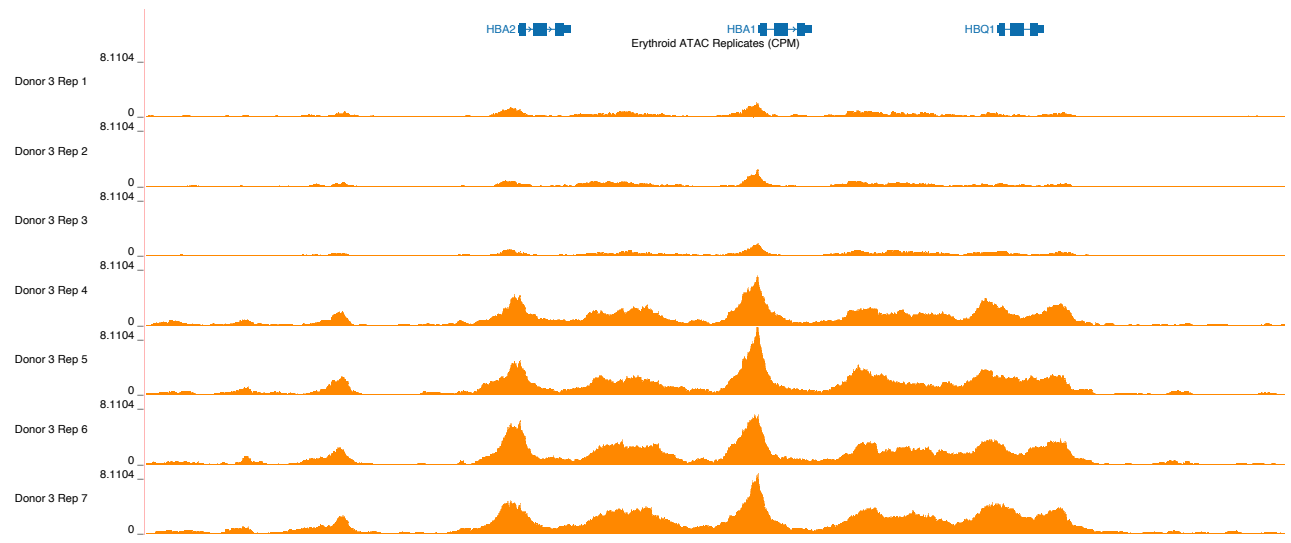

(b) CPM

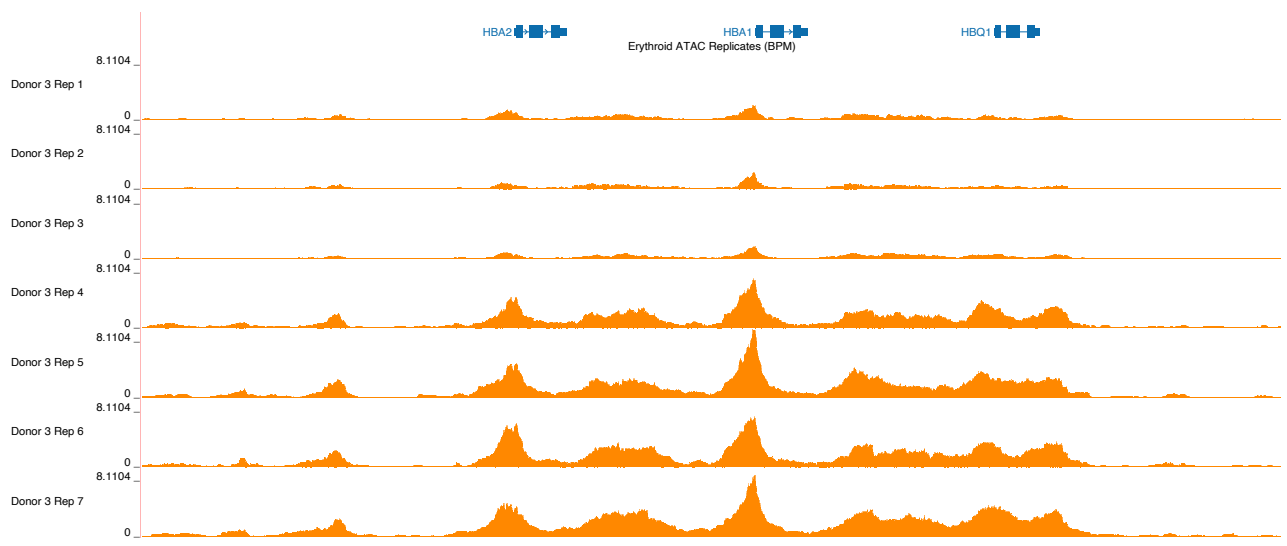

(c) BPM

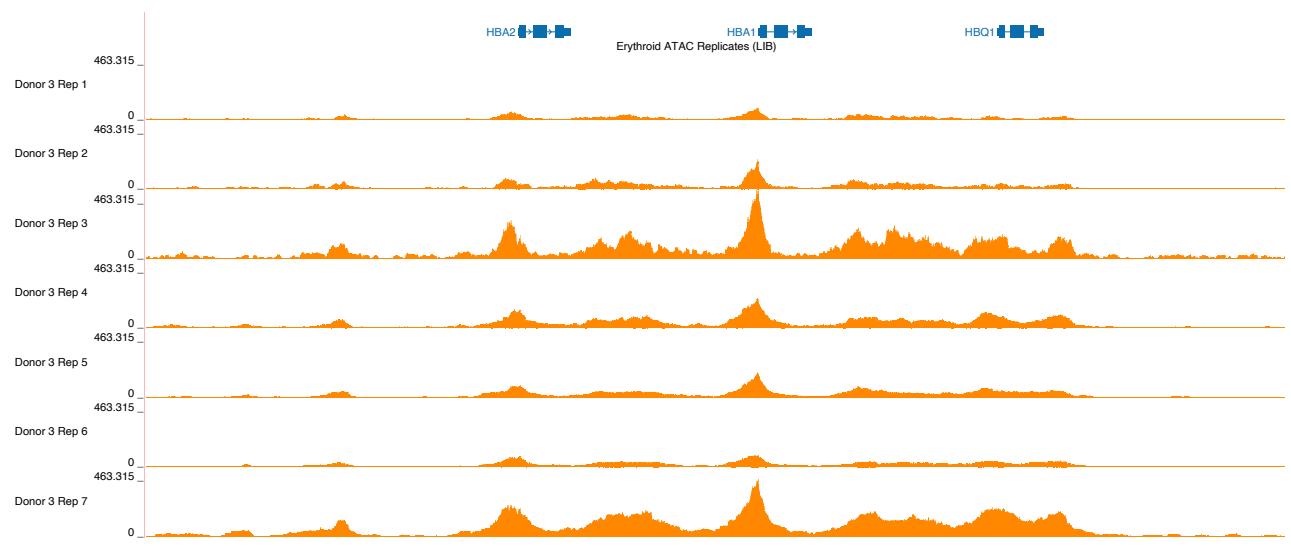

(d) LIB

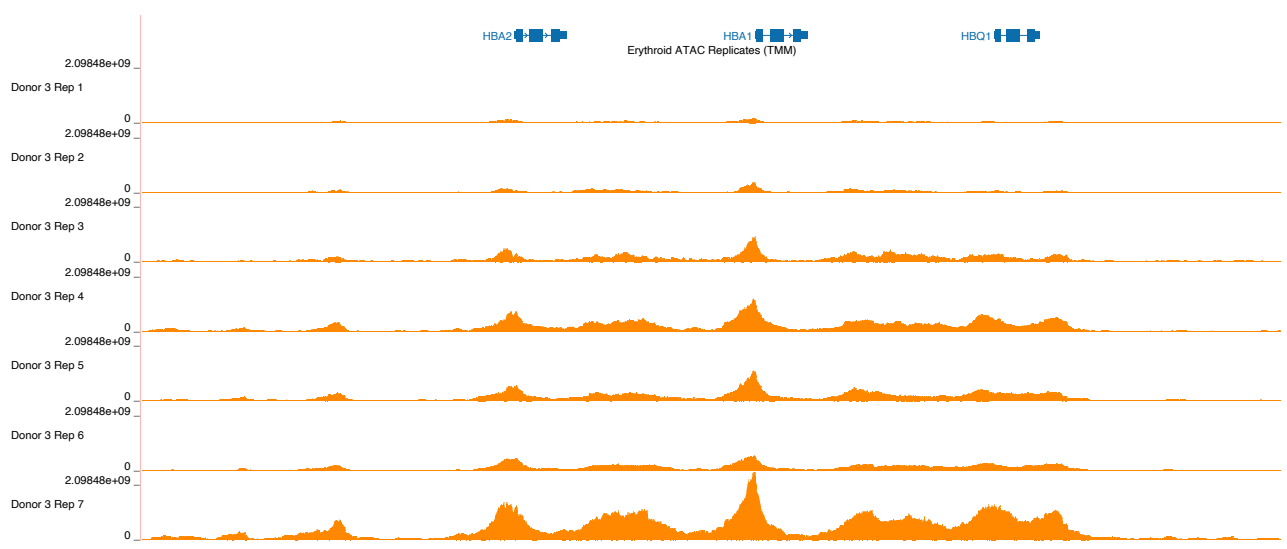

(e) TMM

Figure S8. Genome Browser view of erythroid donor 3 ATAC-seq coverage over chromosome 16 (chr16:167,000-185,000) across the alpha-globin *locus* and surrounding genes (*HBM*, *HBA1*, *HBA2*, *HBQ1*. Tracks show technical replicates 1 to 7 for RPKM (a), CPM (b), BPM (c) LIB (d), and TMM. For no normalisation, RPGC, RLE, and ZEN, see Figure 2.

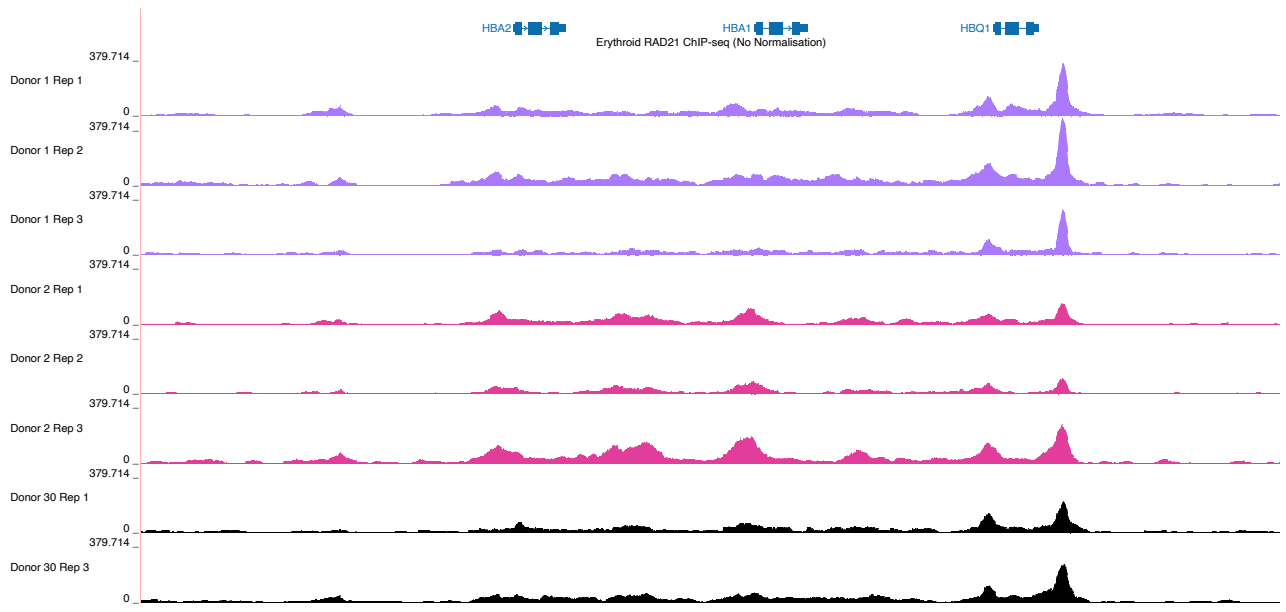

(a) Raw

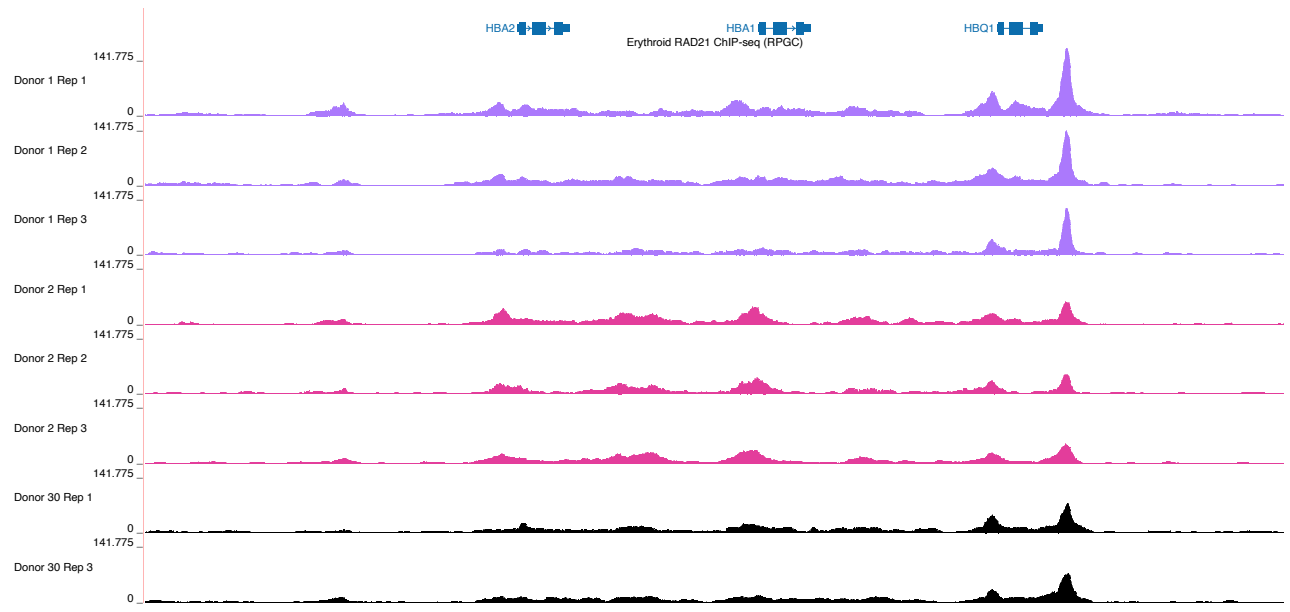

(b) RPGC

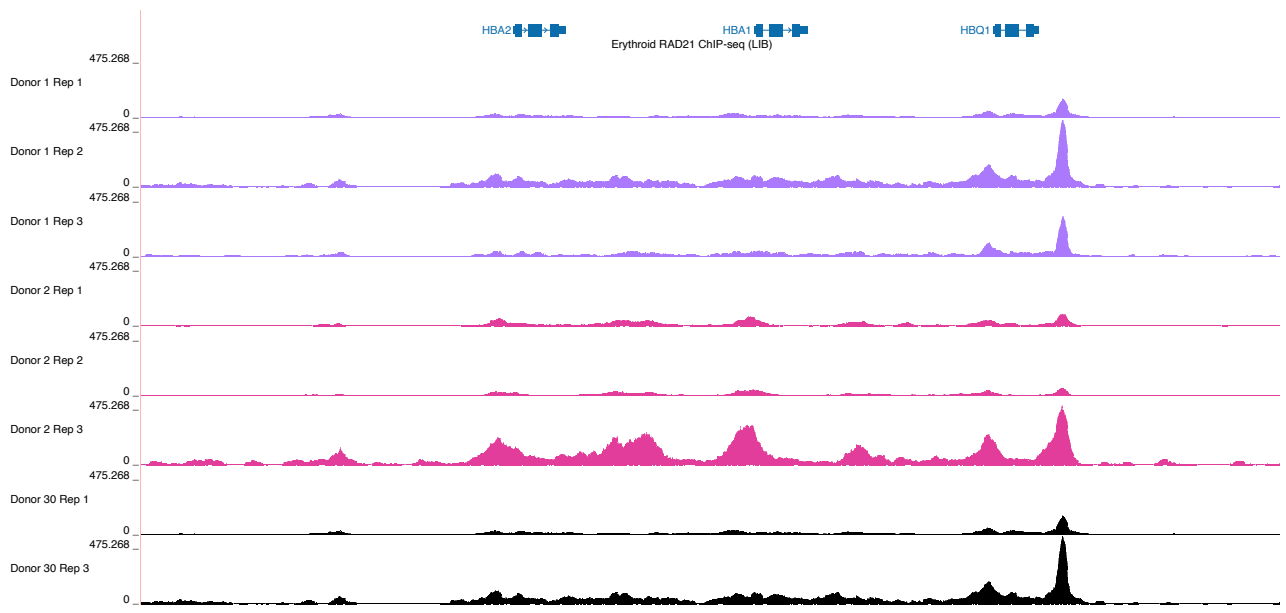

(c) LIB

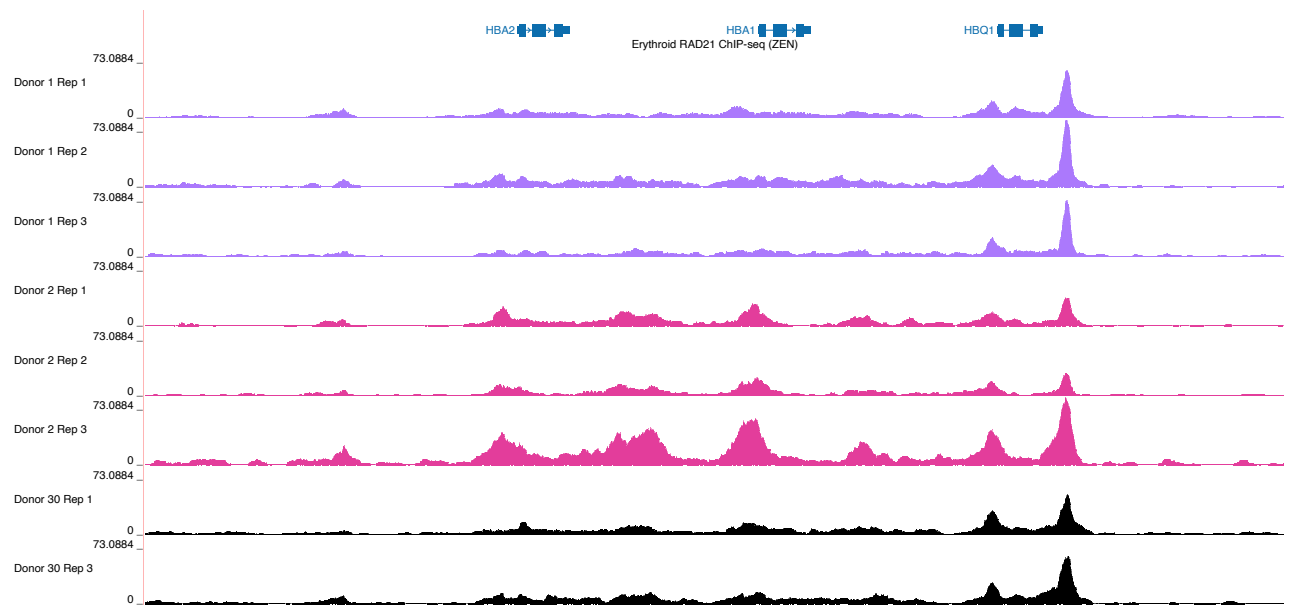

(d) ZEN

Figure S9. Genome Browser view of erythroid donor 2 (purple), 3 (pink) and 30 (black) RAD21 ChIP-seq coverage over chromosome 16 (chr16:167,000-185,000) across the alpha-globin *locus* and surrounding genes (*HBM*, *HBA1*, *HBA2*, and *HBQ1*). Tracks show all technical replicates without normalisation (a), RPGC (b), LIB (c) and ZEN (d).

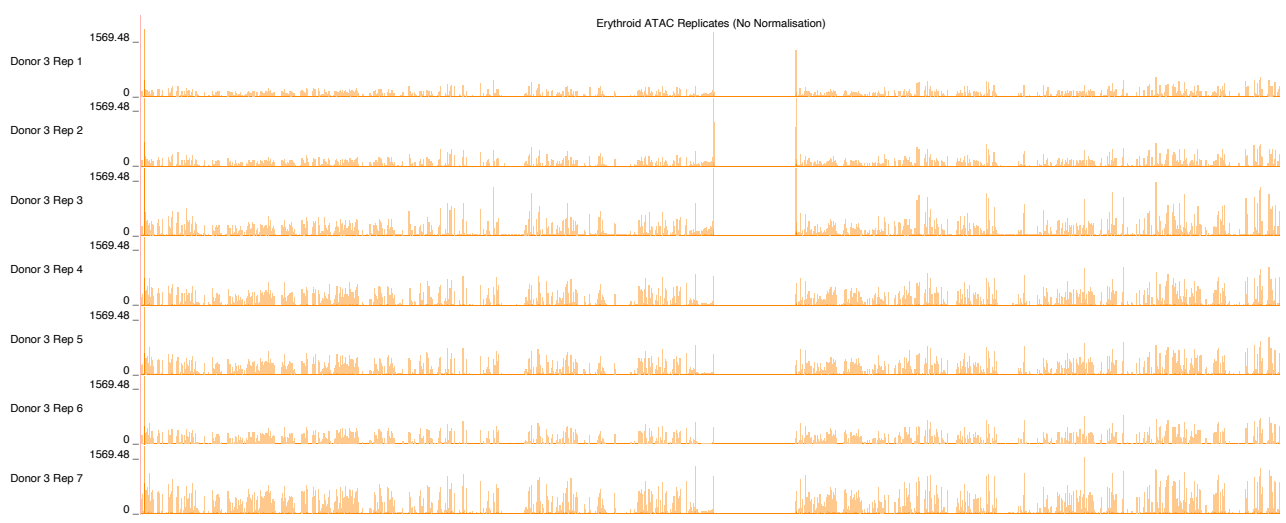

(a) Chromosome 1

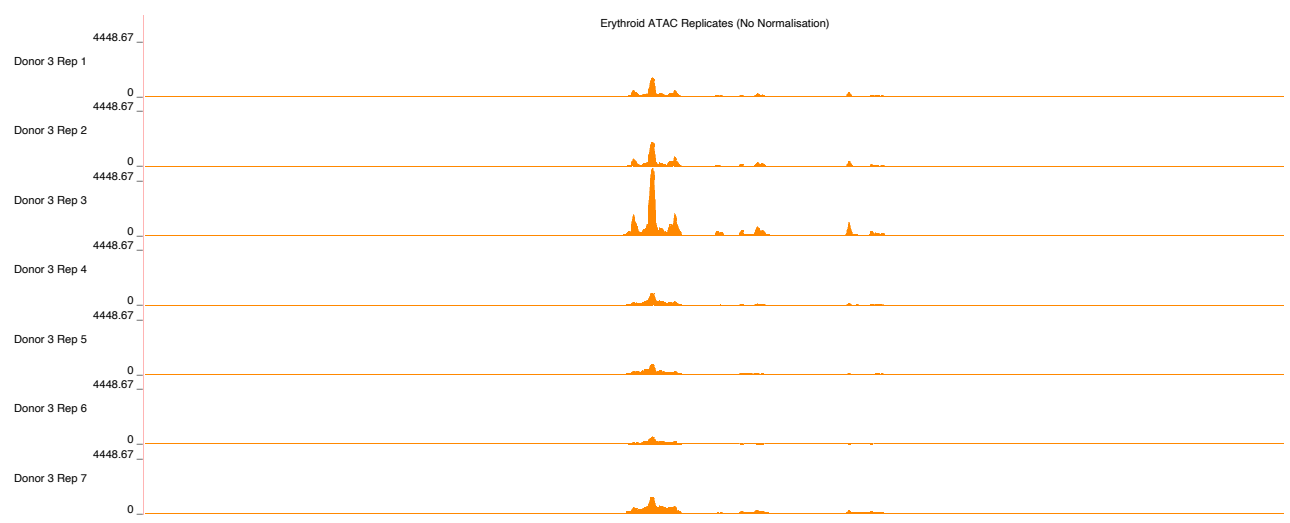

(b) Chromosome 13

Figure S10. Genome browser view of erythroid donor 3 ATAC-seq coverage over chromosome 1 (a) and chromosome 13 (chr13:18,205,000-18,221,000) (b). Both depict non-blacklisted regions where the unnormalised signal is extremely high and variable across technical replicates 1 to 7 near the centromeres. For (a), the full chromosome is show with the artifact towards the centre. In (b), it can be seen that the artifact has far higher magnitude than the surrounding signal.

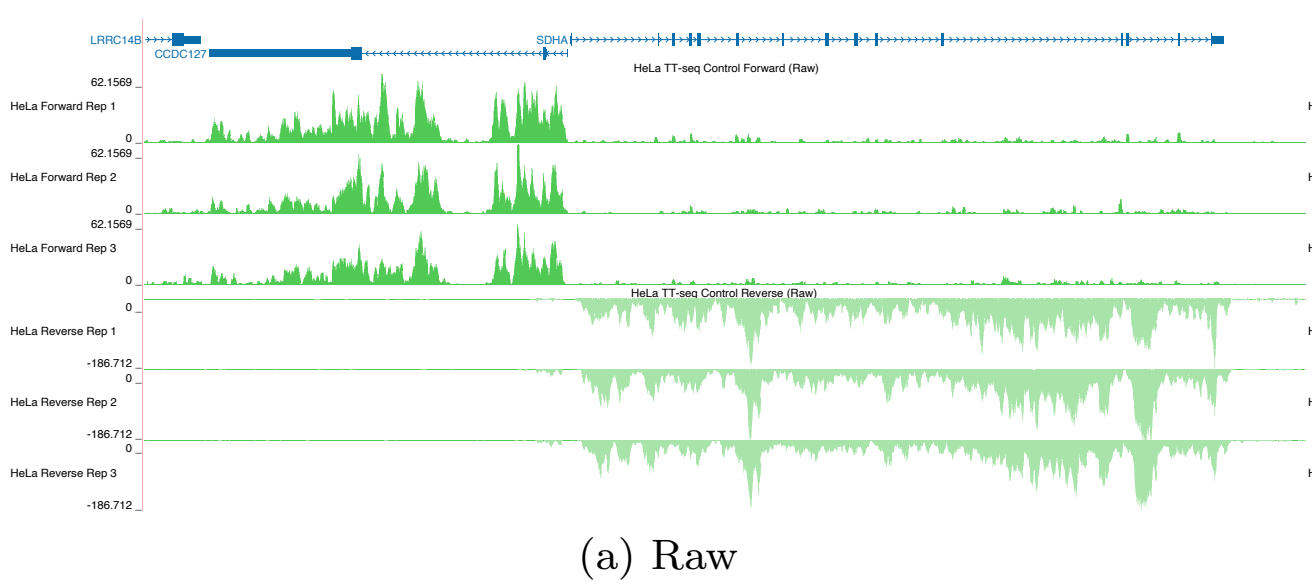

(a) Raw

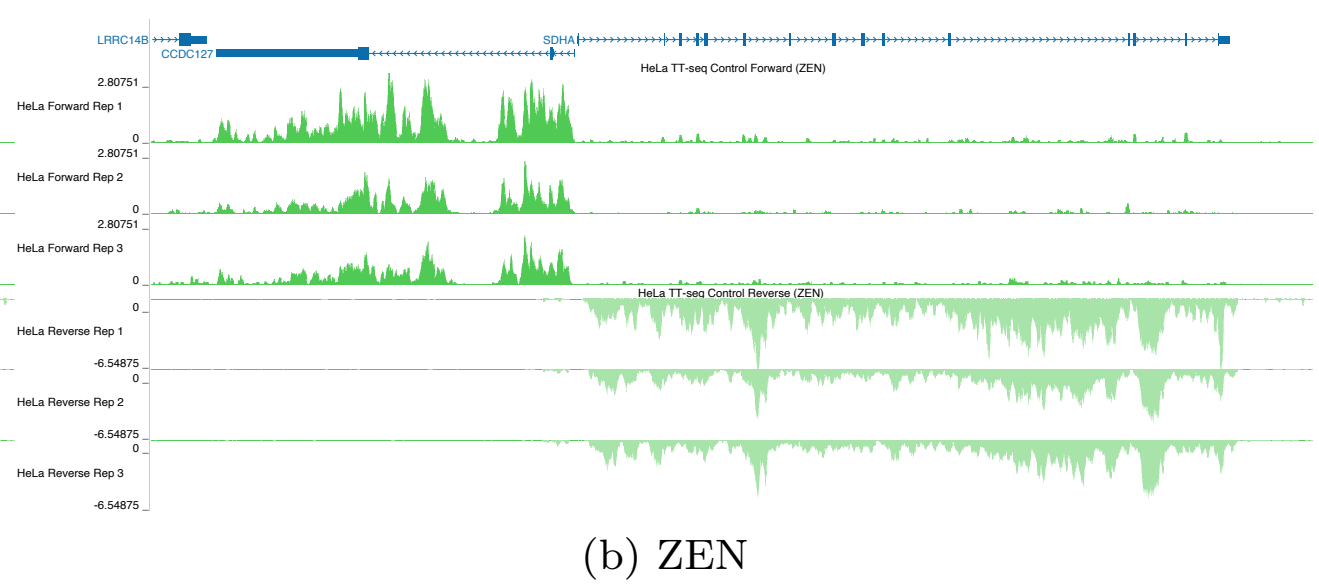

(b) ZEN

Figure S11. Genome Browser view of HeLa TT-seq over chromosome 5 (chr5:193,000-262,000). Forward strand transcription is over *CCDC127* and reverse strand transcription over the housekeeping gene *SDHA*. Tracks show technical replicates 1 to 3 with and without normalisation (a) and ZEN (b).

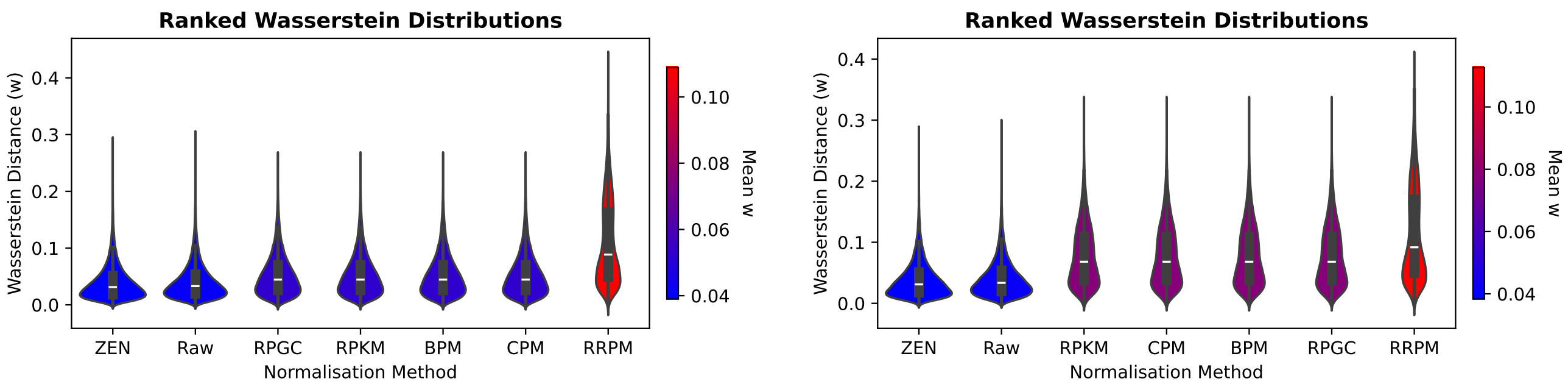

(a) HEK293T TT-seq (Forward Strand)

(b) HEK293T TT-seq (Reverse Strand)

Figure S12. Comparing normalisation methods by min-max scaled Wasserstein distance between sample pairs across signal regions genome-wide. (a) and (b) show forward and reverse strand performance from HEK293T TT-seq with four technical replicates. Signal regions are padded zones (broad regions of transcription). Violin plots are ranked and coloured by mean Wasserstein distance, where a lower distance implies better alignment.

E. DiffBind differential accessibility analysis

E.1. Significantly different erythroid ATAC-seq peaks per normalisation method

Intercept	Comparison	Normalisation	Significant Peaks
Donor 2 replicates 1–3	Donor 2 replicates 4–6	LIB	14,389
		RLE	5,943
		TMM	6,356
		ZEN	14
Donor 2 replicates 1–7	Donor 3 replicates 1–7	LIB	332
		RLE	1,049
		TMM	1,071
		ZEN	145

Table S2. Number of significant differentially accessible peaks between erythroid technical and biological replicates. DA analysis was run with DiffBind and LanceOtron peaks (overall peak score > 0.5). The first four rows show comparisons between all erythroid ATAC-seq donor 2 replicates and donor 3 replicates for LIB normalisation, RLE, TMM and ZEN. The last four rows show results between technical replicates 1 to 3 and 4 to 6.

E.2. Biologically different peaks between erythroid donor 2 and 3

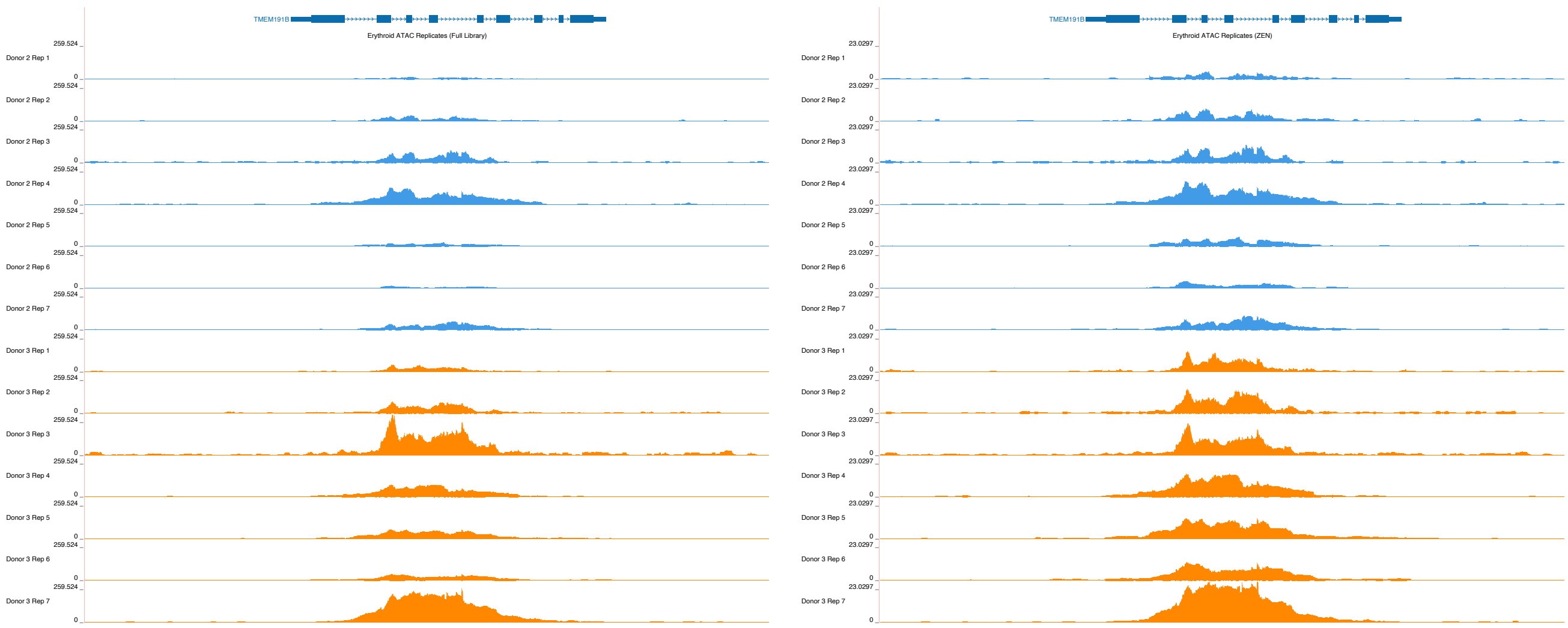

(a) LIB (Default Normalisation) (b) ZEN

Figure S13. Genome Browser view of erythroid donor 2 replicates 1-7 (blue) and donor 3 replicates 1-7 (orange) ATAC-seq coverage over *TMEM191B* (chr8:9,146,000-9,177,000). Accessibility over the gene body is predicted by DiffBind as significantly increased (FDR < 0.05) from donor 2 and 3 by all methods. This difference is easier to verify when replicates are normalised with ZEN (b) compared to LIB (a) as ZEN improves technical replicate consistency.

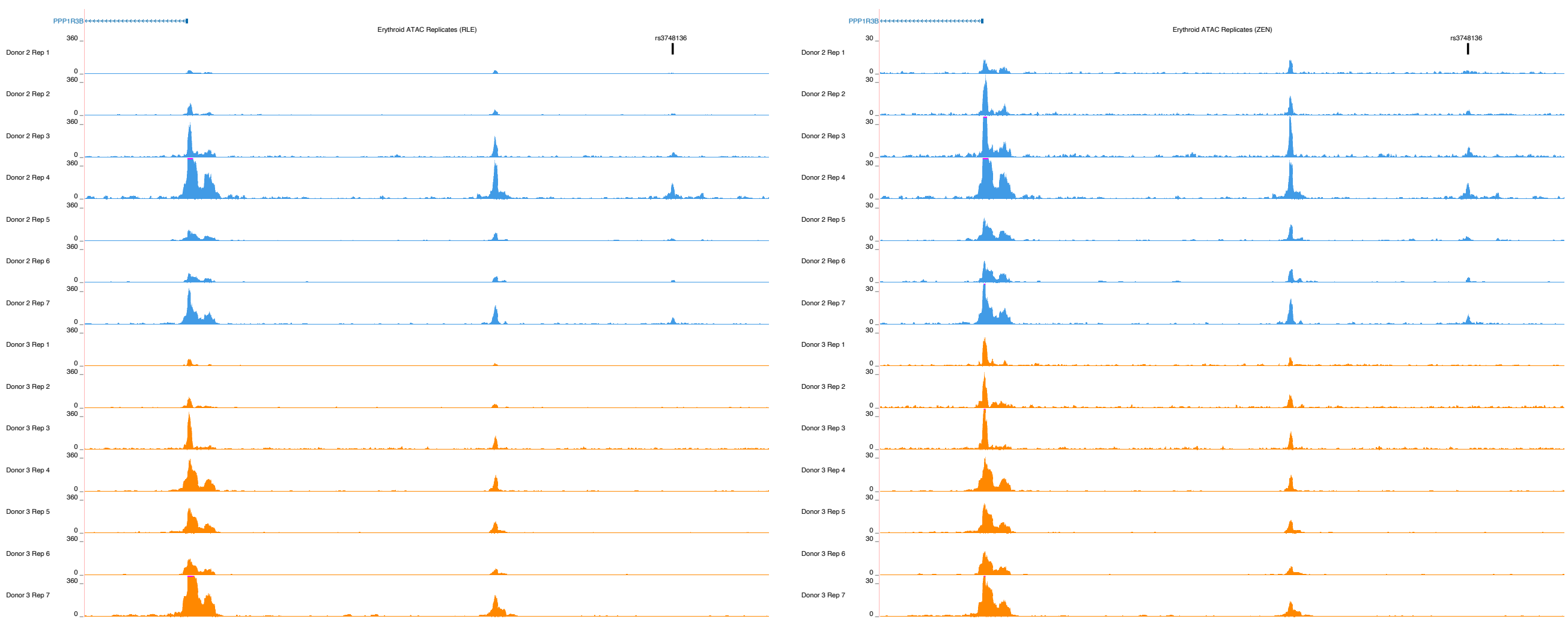

(a) RLE (b) ZEN

Figure S14. Genome Browser view of erythroid donor 2 replicates 1-7 (blue) and donor 3 replicates 1-7 (orange) ATAC-seq coverage of *PPP1R3B* promoter and two downstream enhancers (chr8:9,146,000-9,177,000). The small peak over the right enhancer only occurs in donor 2, due to a gain-of-function SNP rs3748136 (G>A) that was predicted as significantly different by all methods. Reduced variability between technical replicates from the same donor makes this clearer when viewed with ZEN normalisation. The middle enhancer was predicted by DiffBind as a significantly different peak (FDR < 0.05) between donor 2 and 3 when normalised with RLE (a) and TMM, but not with ZEN (b) or LIB.

E.3. False positive peaks between erythroid donor 2 and 3

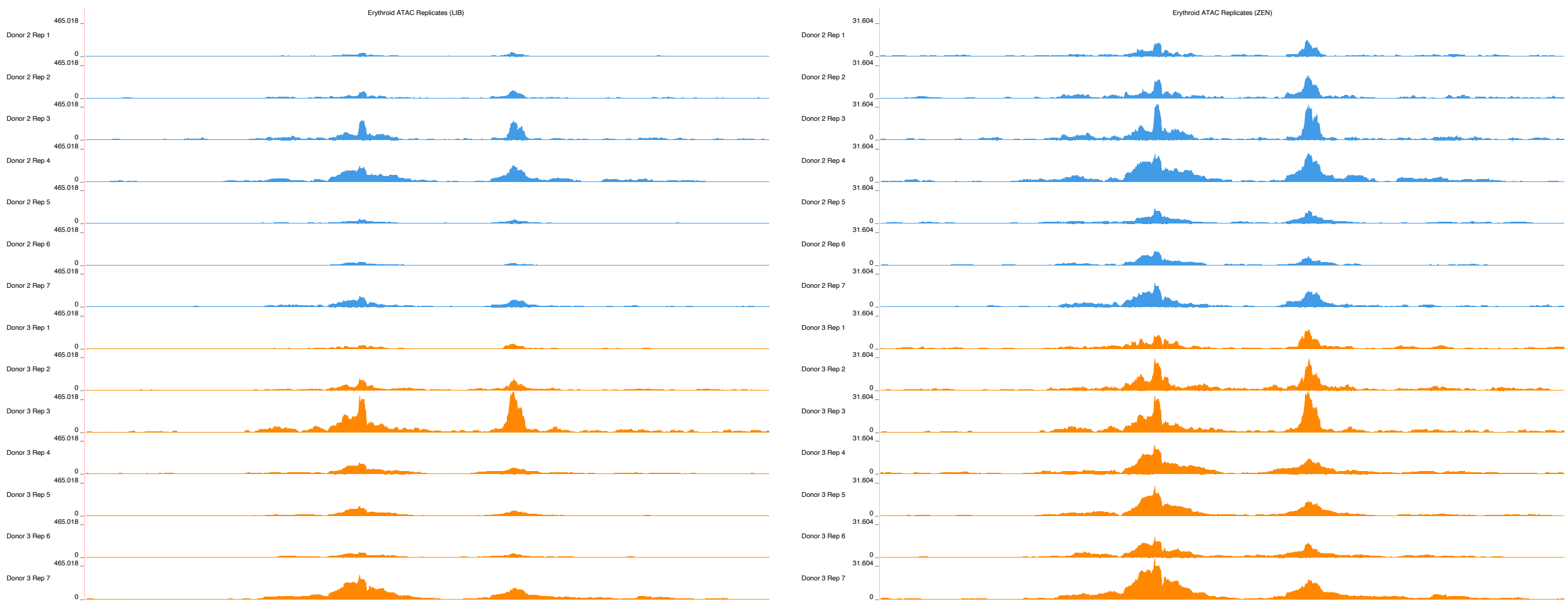

(a) LIB (Default Normalisation) (b) ZEN

Figure S15. Genome Browser view of erythroid donor 2 replicates 1-7 (blue) and donor 3 replicates 1-7 (orange) ATAC-seq coverage over a chromosome 9 non-coding region (chr9:40,103,000-40,112,000). The left LanceOtron peak was predicted by DiffBind as significantly different (FDR < 0.05) between donor 2 and 3 with LIB normalisation (a) RLE and TMM, but not with ZEN (b) as technical variability between replicates is reduced.

References

Akinshin A. The median absolute deviation value of the Gumbel distribution, October 2020. Online article, published 6 October 2020.

Aptekmann AA et al. Transcription factor specificity limits the number of dna-binding motifs. Plos one 2022;17:e0263307.

Benjamini Y, Yekutieli D. The control of the false discovery rate in multiple testing under dependency. Annals of statistics 2001:1165–1188.

Diagenode. Guide for optimal chromatin preparation using Chromatin EasyShear Kits. Diagenode technical guide. Online resource.

Georgiades E et al. Active regulatory elements recruit cohesin to establish cell specific chromatin domains. Scientific Reports 2025;15:11780.

Goodwin S, Wappel R, McCombie WR. 1d genome sequencing on the oxford nanopore minion. Current Protocols in Human Genetics 2017;94:18–11.

Harrell FE, Davis C. A new distribution-free quantile estimator. Biometrika 1982;69:635–640.

Heckert NA et al. Handbook 151: NIST/SEMATECH e-handbook of statistical methods. 2002.

Insco ML et al. Oncogenic cdk13 mutations impede nuclear rna surveillance. Science 2023;380:eabn7625.

Love MI, Huber W, Anders S. Moderated estimation of fold change and dispersion for rna-seq data with deseq2. Genome Biology 2014;15:550.

Lun AT, Smyth GK. csaw: a bioconductor package for differential binding analysis of chip-seq data using sliding windows. Nucleic acids research 2015;44:e45.

Virtanen P et al. Scipy 1.0: fundamental algorithms for scientific computing in python. Nature Methods 2020;17:261–272.

Yan F et al. From reads to insight: a hitchhiker's guide to atac-seq data analysis. Genome Biology 2020;21:22.

Zhang K et al. A single-cell atlas of chromatin accessibility in the human genome. Cell 2021;184:5985–6001.

Zhang Y et al. Model-based analysis of chip-seq (macs). Genome Biology 2008;9:R137.
